# Design of Surfactant Molecules Under Performance Constraints

**DOI:** 10.1021/acssuschemeng.5c04112

**Published:** 2025-08-20

**Authors:** Sofía González-Núñez, Zeynep Sumer, Carlos Amador, Prakash Madhav, Ajay Muralidharan, Claire S. Adjiman, Mariano Martín

**Affiliations:** a Departamento de Ingeniería Química, 16779 Universidad de Salamanca, Pza. Caídos 1-5, Salamanca 37008, Spain; b Department of Chemical Engineering, Sargent Centre for Process Systems Engineering, Institute for Molecular Science and Engineering, 4615 Imperial College London, South Kensington Campus, London SW7 2AZ, United Kingdom; c Newcastle Innovative Centre, Procter and Gamble, Whitley Rd, Longbenton, Newcastle Upon Tyne, Tyne And Wear NE12 9SR, England; d Mason Business and Innovation Center, 114590 Procter and Gamble, Mason-Montgomery Rd, Mason, Ohio 45040, United States

**Keywords:** computer-aided molecular design, multiobjective optimization, property prediction models, molecular graphs, sustainability

## Abstract

The industrial applications of surfactant solutions are both numerous and extremely diverse, demonstrating the importance of these systems in everyday life and driving the need for a systematic approach to designing sustainable surfactant molecules adapted to the specific requirements of each application. Given the very large space of possible molecules, the identification of candidate surfactants that achieve a balance between the optimal physicochemical properties of the product and minimal environmental and health impacts is extremely challenging. In this work, a formulation and solution framework based on Computer-Aided Molecular Design is proposed for surfactant design. A novel multistage methodology is developed based on the initial generation of promising candidates for the two constituents of a surfactant, the hydrophilic head and the hydrophobic tail, followed by the multiobjective optimization of surfactant molecules. This decomposition results in an effective solution strategy. In addition to constraints that ensure the generation of feasible molecules, specific structural constraints can be incorporated in the formulation, accelerating the discovery and optimization process. Data-driven predictive models for the most relevant surfactant properties, such as critical micelle concentration, Krafft point, surface tension, toxicity, and biodegradability, are developed and implemented in the optimization formulation. Two case studies are tackled, successfully generating novel surfactant molecules. The proposed framework could be extended to more complex structures, such as two-headed or Gemini surfactants.

## Graphical abstract

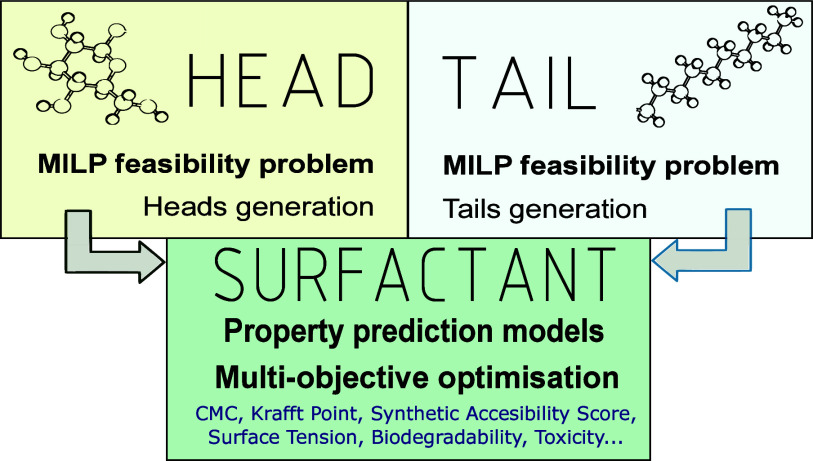

## Introduction

In recent decades, the growing awareness of environmental issues has prompted industry to seek sustainable alternatives for conventional chemical processes and products. In this context, the development of computer-aided design methods for molecular design,^1^ and the recent integration of artificial intelligence (AI) and machine learning (ML) models in this framework, has demonstrated remarkable capabilities to monitor, analyze and optimize complex systems to aid data-driven decision-making.^2^ Among the revolutionary innovations that hold great promise for addressing pressing environmental challenges are ″designer molecules″. Designer molecules should in principle be engineered to meet a range of criteria based on their performance in *a specific application* and on their impacts on health, safety and the environment throughout their life cycle, from raw materials to production, use and eventual disposal. Design methods that bring together the principles of sustainability and molecular engineering^3,4^ have the potential to help us address the pressing challenge of more sustainable production and consumption.^5^ This vision is however not trivial to realize due to the existence of conflicting design criteria, the vast number of possible designs and the complexity of the relationships between the structure of a molecule and its performance, technical or otherwise. Surfactants, with their complex influence on product performance and their use in products that are often destined to enter waste streams (e.g., cleaning products), offer a worthwhile challenge to advance design methods.

Surfactants, short for surface-active agents, are compounds that have the ability to reduce the interfacial tension between two phases. They are made up of two constituent parts, a hydrophobic (water-repelling) and a hydrophilic (water-attracting) part, which allow them to adsorb at the interface between two separate phases, such as air and water. This reduces the interfacial tension between the two phases, enabling them to form stable emulsions.^6^ As a result, surfactants are widely used in a range of applications, including personal care products, detergents, and agricultural chemicals. However, traditional surfactants, which are commonly derived from nonrenewable petrochemical sources, can pose challenges related to biodegradability and aquatic toxicity. When released into the environment via wastewater, some surfactants can persist and accumulate, causing water pollution and disrupting delicate aquatic ecosystems.^7,8^

There is a need to produce or select alternative surfactants with lower environmental impact. Among the alternative surfactants, there has been a growing interest in the design of biosurfactants, which are derived from renewable resources and are designed to be biodegradable and nontoxic. These properties have allowed the use of biosurfactants in numerous and diverse applications including waste remediation through the removal of inorganic substances, such as heavy metals,^9^ and of organic compounds, such as hydrocarbons;^10^ or the elimination of water from emulsions prior to processing,^11^ making them valuable for oil recovery.^12,13^ Furthermore, biosurfactants are increasingly used and preferred in the food industry and in the health and cosmetic sectors. This makes it important to be able to design novel surfactant structures or to select surfactants that are tailored to the specific application of interest. The need to identify different surfactants for different applications motivates the use of “digital-first” design approaches that integrate modeling and computational tools to predict relevant properties and key performance indicators (KPIs). In this context, computer-aided molecular design (CAMD) has emerged as a powerful tool for the generation of structures with desirable properties in a variety of fields, including materials science or drug design.^14^ By using computational methods to predict the properties of molecules, CAMD enables the rapid screening and optimization of large numbers of candidate structures, thereby accelerating the discovery process. Importantly, it also allows for the integration of key environmental performance criteria such as biodegradability and aquatic toxicity directly into the molecular design and selection process. Although this approach does not explicitly constrain the search space to molecules of renewable origin, it enables the systematic prioritization of novel candidates with low environmental impact, an essential objective in the development of improved (bio)­surfactants. In recent years, significant progress has been made in the development of CAMD methods, including techniques for *de novo* molecule design and property-based optimization, using first-principles models and/or machine learning-based approaches.^15–17^ Of relevance to surfactant design is the advent of methods for the design of mixtures or blends (often referred to as CAM^b^D), since surfactants are always used within a mixture and often formulated using two or more compounds.^18,19^

The design of surfactants with desired properties, such as low critical micelle concentration (CMC), a high efficiency in surface and interfacial tension reduction and biodegradability, is a complex task that requires knowledge of the structure–property relationships of the compounds involved.^20,21^ The optimization of the molecular structure is of vital importance to achieve the desired characteristics in the surfactant to be produced. For instance, the CMC is an essential property that governs the self-assembly behavior of surfactants, which is itself strongly influenced by the molecular structure of the surfactant (such as the tail length and the head area). It is typically observed that the shorter the hydrophobic tail and the larger the hydrophilic head, the higher the CMC.^6,22^ A further challenge for CAMD is that surfactant molecules are often comparatively large, featuring hydrocarbon tails generally between 10 and 18 carbons, as well as heads that can also be large, with groups such as glucose or maltose. As a result, surfactant molecules have a wide range of molecular weights, spanning from 170 to 1300 Da. This is much larger than what has been considered in many CAMD studies for solvents^23–26^ or heat transfer fluids^27,28^ and therefore poses a much greater combinatorial challenge.

Despite these challenges, promising CAMD approaches for surfactant design have emerged. Cheng et al.^20^ utilized CAMD techniques to optimize nonionic surfactants when combined with the widely used anionic surfactant, SDS, to achieve desired detergent properties. The design of the surfactant structure was framed as a mixed integer nonlinear program (MINLP) and group contribution methods (GCMs) were applied to estimate key properties. A multiobjective optimization problem was formulated and solved using the ε-constraint method,^29^ with CMC as the primary objective and cloud point (CP), hydrophilic–lipophilic balance (HLB) and molecular weight (MW) as secondary objectives (i.e., handled via the ε constraints). In the study by Liu et al.,^30^ a case study based on the works of Mattei et al.^31,32^ and Seider et al.^33^ was tackled with the objective of designing a surfactant with the lowest value of a toxicity metric. A CAMD-based MINLP problem was formulated and GCMs were used, with the introduction of additional structural constraints such as the total number of groups (including functional groups) comprising the surfactant molecule to further refine the design process. Alshehri et al.^34^ addressed the limitations of relying solely on GCM-based approaches (which they refer to as GC-Simplemethods) by developing GC_ML methods that achieve better prediction statistics and deploying these in the solution of CAMD problems previously tackled with GCMs, including the aforementioned surfactant design problem of Liu et al.^30^ Specifically, they use Gaussian Process Regression models.

Consequently, studies on quantitative structure–property relationships (QSPRs) and understanding of the effects of molecular structures on their functions and properties are becoming increasingly important in surfactant design. A multitude of molecular thermodynamic theories have emerged to elucidate the surface behavior and CMC of surfactant systems.^35–39^ Self-consistent field (SCF)^40,41^ and single-chain mean field theories have been employed to investigate CMC phenomena.^42,43^ Atomistic molecular simulations have significantly enhanced our molecular-level understanding of interfacial surfactant behavior, with some simulations being used to provide insights into molecular insights.^44^ However, these use of such simulations in design poses overcoming challenges attributed to the extensive system sizes and computational cost. Recent advances also encompass molecular equations of state and density functional theory techniques,^45,46^ which are less computationally demanding. However, such theoretical models are limited to bulk thermodynamic properties or semiquantitative investigations of surfactants with relatively uncomplicated chemical structures. These computational and theoretical approaches are far from being systematically deployable across a wide range of amphiphiles and from being used to generate high-quality design data. Given the complexity of the structure–property relationships that characterize surfactant behavior the recent emergence of ML models to predict a broad range of properties,^47^ including properties of direct relevance to surfactant performance,^21^ has been encouraging. It is the ability to systematically discover relevant molecular descriptors based solely on the analysis of the relationships between inputs (descriptors) and outputs (target properties) that positions the hybrid approach of developing ML models and quantitative structure–property relations (QSPR) as a promising path to the correlation and prediction of thermophysical properties for use in CAMD.

Given this context, we address two key challenges: (i) a computational challenge by proposing a decomposition strategy to formulate and solve the surfactant design problem, and (ii) a modeling challenge for hard to predict properties, where we aim to design predictive models that offer a balance between accuracy, transferability and tractability (i.e., ease of use within optimization).

To address the computational challenges associated with the size of surfactant molecules, we propose a decomposition strategy based on the generation of promising candidates for the two constituent parts, head and tail, comprising the surfactant, followed by the optimization of the combined structure, by selecting the best combination(s) of head and tail from the lists of candidates. This modular strategy represents a significant advancement in molecular design, complementing traditional monolithic CAMD formulations that integrate structure generation and property optimization into a single, tightly coupled MINLP problem. A key advantage of these one-step approaches is that property evaluations are confined to a subset of candidate molecules explored by the optimizer, enhancing computational efficiency. While effective for designing small and relatively simple molecules, this tight coupling generally restricts the property prediction methods that can be used to group contribution approaches, which cannot easily handle the mathematical complexity of advanced ML-based property predictions required for larger, architecturally complex systems like surfactants. This work builds upon the growing body of molecular design research incorporating ML techniques, such as genetic algorithms applied to molecular fuel design^48^ and modular graph-ML in CAMD workflows.^49^ Extending this promising paradigm to surfactant design, our approach leverages modularity and data-driven modeling to broaden the accessible chemical space. In particular, by designing head and tail fragments separately, our approach reduces the size of the feasible region (i.e.,the number of possible discrete variable combinations) thereby enabling the exploration of structurally diverse and chemically relevant candidates. This decomposition strategy builds on the MILP-based model developed by Samudra and Sahinidis,^50^ which demonstrated orders-of-magnitude improvements in computational efficiency over traditional MINLP formulations. The approach effectively mitigates the combinatorial explosion inherent to the design of large molecules, where the rapid growth in structural possibilities, combined with numerous integer variables and nonlinear property models, can result in significant computational and solver challenges. As a result, this modularity reduces the computational time required while giving flexibility in imposing restrictions and desired attributes for the intended surfactant molecule (e.g., number of different molecular groups in the head, minimum number of carbons in the tail^45,46^ ). Being able to address this type of molecular structure expands the relevance of CAMD since there are few studies on the *de novo* design of two-headed surfactants.^51,52^

To address the modeling challenge, several property prediction models are developed. While CAMD methods are often based on using a specific definition of molecules and an associated class of property models, for instance using functional groups^53^ and group contribution methods (GCMs),^54^ or molecular connectivity indices^55–57^ here we make use of quantitative structure–property relations based on molecular descriptors,^22,54,58^ which offer relative mathematical simplicity and good accuracy in the prediction of properties governed by complex, nonlinear structure–property relationships. The integration of this QSPR framework within the proposed decomposition approach constitutes a flexible and extensible methodology for early stage surfactant design.

In the remainder of this paper, the decomposition-based methodology is presented in detail, beginning with the generation of head and tail structures. This is followed by an exposition of the multiobjective surfactant design problem and of the strategy adopted to develop the relevant property prediction models. The implementation of the framework is discussed briefly. The performance of the property prediction models is examined in the Results section and the framework is deployed on two case studies to investigate the applicability of the proposed approach.

## Methodology

The proposed methodology addresses the challenges faced by CAMD methods in surfactant design by dividing the problem into two main phases: 1) generation of head and tail candidates that can be combined into surfactant molecules that meet with specified structural requirements, and 2) multiobjective optimization of surfactant properties. The overall workflow is shown in Figure 1.

**Figure 1:**
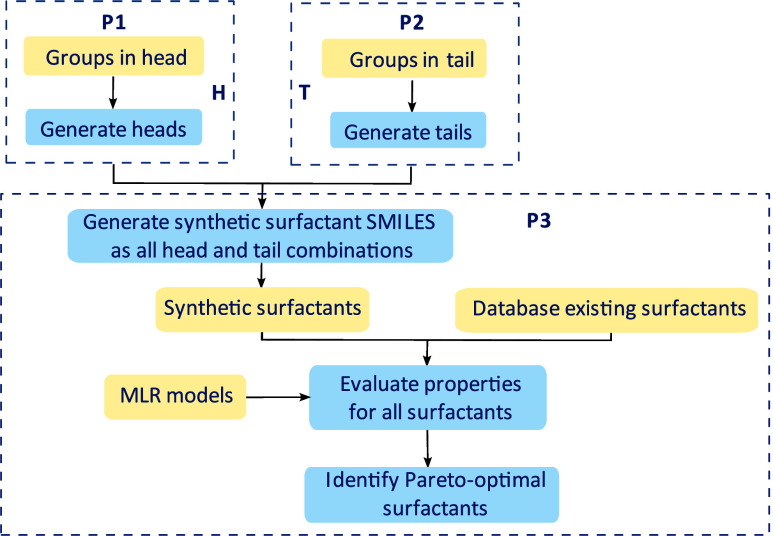
Overall workflow for surfactant design. Yellow corresponds to input information and blue to actions. The acronym MLR corresponds to multiple linear regression.

### Head and Tail Generation

To generate a suitable set of heads and tails, two CAMD subproblems are formulated (P1 for heads and P2 for tails, in Figure 1). For each subproblem, a set of functional groups is defined together with rules that specify how the groups can be connected to form a meaningful head or tail. In addition, desired structural molecular constraints can be incorporated into each subproblem. To develop a mathematical representation for these problems, a graph-based representation of molecules is adopted wherein the nodes correspond to groups in the molecule and the edges define the bonds within the molecule, i.e., the connectivity between groups.^50^

### Definition of a Database of Groups

The chemical space is defined for the particular case of surfactants, wherein groups are split according to the constituent part they can belong to hydrophobic tail, hydrophilic head, or both. For this purpose, a fragmentation and assignment of basic molecular groups present in surfactants available in the literature is carried out, covering as much molecular space as possible. Typical examples of these groups are O = S­(=O)­[O-] or [CH2]­[NH] for the head, aromatic carbons represented by lower case c for the tail, and aliphatic carbons represented by upper case C for both parts. The specific groups considered for each case study are presented in the Results section.

### Adjacency and Bond Matrices

The adjacency matrix, in which each row and each column correspond to nodes (atom groups) in the molecular structure, is a way of representing a graph as a square matrix of ones and zeros. This representation has the advantage of simplicity but it does not allow the description of different bond types (single, double, triple). To preserve this simplicity while allowing for more diversity in the bond type, all connections between nodes denote single bonds and the groups are defined such that any double or triple bonds appear within atom groups only, e.g., −CH = CH_2_ appears as a group that can be linked to any other group via a single bond. Focusing on single-bond connections between nodes simplifies the CAMD problem formulation as fewer constraints are required to avoid the generation of unrealistic molecular groups. Once a head or tail is generated in the form of an adjacency matrix, the representation of each node can be expanded via bond matrices. In a bond matrix, each row and each column correspond to an atom in the atom group. The entries in the bond matrix represent the type or order of the bond between the atoms (2 for double, 3 for triple, etc.), and not only the existence of a bond (0 or 1) as in the adjacency matrix. This more detailed description can form the basis for the generation of molecular descriptors.

### Graph Generation Model (MILP)

Two graph generation models are formulated as mixed integer linear programs (MILPs) to generate structures for heads and tails. A binary variable, denoted as *y*_*jj*′_, is used to model the presence (*y*_*jj*′_ = 1) or absence (*y*_*jj*′_ = 0) of a bond between each pair of nodes *j* and *j*′. The basic formulation used for generating graphs of molecular structures is that presented by Samudra & Sahinidis.^50^ In this model, J represents the set of groups used as model inputs, $J_{0}$ represents the set of groups with free valence (allowable bonds) equal to 1, and thus, $J\backslash J_{0}$ refers to the set of groups with valence greater than 1. This distinction is made because in the graph, all single valence groups of one type, such as – CH_3_ are collapsed into the same node. For example, if there are four −CH_3_ groups, a single node *j* with total valence *v*_*j*_ = 4 is used to eliminate many redundant solutions. As an example, the graph representation and the adjacency matrix of the 1,4-dioxo-1,4-dipentoxybutane-2-sulfonate molecule are shown in Figure 2. The graph is seen to contain a node with two CH_3_ groups. The term ″node″ is thus used generically because, although in most cases a node represents a single group, there are also cases where a node represents several groups of the same type. Nonetheless, the model allows specifying the maximum occurrences of any molecular group in the structure by defining the parameter *n*_*j*_ as an input, which sets this upper limit. Equation 1 incorporates additional instances into the set of groups J as

$$J\leftarrow J\cup \left\{j_{b}|b\in \left[1,n_{j}-1\right]\right\},\mathrm{if}\;n_{j}\neq 1,\forall j\in J$$ 1

**Figure 2:**
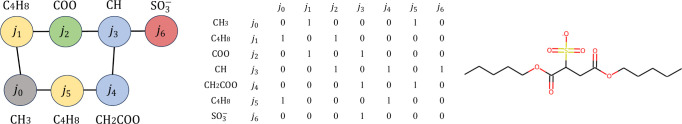
Graph, adjacency matrix and molecular representation of 1,4-dioxo-1,4-dipentoxybutane-2-sulfonate. The CH_3_ group is seen to be connected to two groups, indicating that two CH_3_ groups are in fact present in the molecule.

All remaining inputs, including valencies and atomic connections, are duplicated accordingly. For instance, two C_4_H_8_ units appear in the structure (*n*_C _4_ H_8__= 2). Therefore, each group with a valence greater than one is considered an independent node, even if multiple groups of the same type are present. The example also brings up a limitation of the representation used: the CH_2_COO group is asymmetric so that either the alkyl carbon or the ester carbon could be connected to the CH group. In the presence of asymmetric groups, some isomers are represented by the same adjacency matrix.

The graph generation model is formulated using graph optimization techniques and solved repeatedly, adding uniqueness and redundancy cuts for each structure found at each iteration, to avoid generating duplicate structures. Furthermore, it is worth noting that with the additional structural constraints that can be included, the generation of structures that are not relevant to surfactant design may be avoided. The model is described next and is implemented in Python Optimization Modeling Objects^59^ (PYOMO) and solved using CPLEX.^60^

Since the subproblem being solved is focused on generating all feasible head or tail structures, a dummy objective function is used:

$$\underset{y}{\min}f=0$$ 2

The first constraint ensures that the valence of each group *j* is satisfied:

$$\underset{\begin{array}{c} j^{\prime }\neq j\\ j^{\prime }\in J \end{array}}{\sum }y_{jj\prime }=v_{j}\forall j\in J$$ 3

Equation 4 ensures the symmetry of the adjacency matrix.

$$y_{jj\prime }=y_{j\prime j}\forall j\in J,j^{\prime }\in J$$ 4

Equation 5 implies that two groups with valence greater than one can only be connected to each other by a single bond (recall that double/triple bonds are included within each node, for which the bond matrix is used).

$$y_{jj\prime }\leq 1\forall j\in J\backslash J_{0},j^{\prime }\in J\backslash J_{0},j^{\prime }\neq j$$ 5

Equation 6 ensures that a group cannot form a bond with itself:

$$y_{jj}=0\forall j\epsilon J$$ 6

Note that this also precludes molecules with two identical, single valence groups (e.g., ethane, CH_3_–CH_3_), but such constructs are not relevant to surfactant design. The fulfilment of the constraints introduced so far does not guarantee that the molecular structures formed are fully bonded graphs, and can result in structures with disconnected components, which are not of interest. To avoid this problem, not all the bonds from multivalence nodes can be used with single-valence nodes. This is represented by eq 7, which guarantees that no disconnected tree components are formed, such as a star graph:

$$y_{jj\prime }\leq v_{j}-1\forall j\epsilon J\backslash J_{0},j^{\prime }\epsilon J_{0}$$ 7

Although necessary, eq 7 is not sufficient and needs to be complemented by other constraints. Equation 8 provides a set of connectivity constraints to ensure that no cycle or tree is formed in any nonempty graph Ĵ of size |Ĵ| less than the maximum subset size $J_{m}=\lceil \frac{|J\backslash J_{0}|+1}{2}\rceil$ :

∑∀j∈J^,j′∈Ĵyjj′≤2|Ĵ|−1,∀Ĵ⊂(φ(J\J0)\Ø),|Ĵ|≤⌈|J\J0|+12⌉ 8

In this equation, the powerset $\varphi \left(JJ_{0}\right)$ refers to the set of all possible combinations of groups with valence greater than one not including the empty set Ø. The maximum number of nodes in the graph is 2|Ĵ| due to the bidirectionality of the connections (the matrix is symmetric as defined in eq 4). The number of resulting constraints *N*_*c*_ is given by

$$N_{c}=\overset{J_{m}}{\underset{i=1}{\sum }}\frac{\left|J\backslash J_{0}\right|!}{i!\left(\left|J\backslash J_{0}\right|-i\right)!}$$ 9

For example, according to eq 8, if the number of groups with valence greater than one is 5, the maximum size of the subset is 3 (|J^|≤Jm=⌈|5|+12⌉=3) . Therefore, the number *N*_*c*_ of connectivity constraints for this small problem is equal to 25.

A further constraint is added to the Samudra and Sahinidis^50^ model in order to ensure that all groups of each subset remain interconnected. This follows from the properties of tree-like structures, where the minimum number of edges required for full connectivity is one less than the number of nodes. Working with symmetric adjacency matrices, the number of connections corresponds to twice the number of edges, due to the bidirectionality of such connections. While this equation guarantees that all groups are included in the structure, it does not necessarily ensure that they form a fully connected graph.

$$\left[\sum_{\forall j\in J,j^{\prime }\in J}y_{jj\prime }\geq 2(|J|-1)\right]$$ 10

To eliminate previously found solutions and prevent repetition during the optimization process, we incorporate a for loop within each feasibility problem, adding integer cut constraints at every iteration. Let *S*_*k*_ denote the set of binary variables *y*_*jj*′_ associated with the k-th previously found solution. Then, for each such solution the following constraint is added:

$$\left[\sum_{\forall j\in S_{k},j^{\prime }\in S_{k}}y_{jj\prime }\leq |S_{k}|-1\right],k\epsilon \{1,\cdot \cdot \cdot ,N_{sol}\}$$ 11

where *N*_*sol*_ is the number of solutions found so far. Equation 11 ensures the solution of the current problem differs from the k-th solution int at least one of the binary variables, thereby excluding *S*_*k*_ from the feasible space in subsequent iterations.

The complete model, eqs 2–11, is formulated twice, once for the generation of the heads and once for the generation of the tails. The models differ in the set of groups as well as in the size restrictions, separately defining the minimum and maximum number of total groups that can form the head and the tail (e.g., it can be specified for heads that the powerset be calculated only for subsets of between 2 and 4 groups instead of for all possible combinations, with tails typically being larger). A breadth-first search (BFS) algorithm^61,62^ is employed to evaluate the connectivity of adjacency matrixes obtained as solutions, initiating from an arbitrary node and traversing all accessible nodes. If the traversal reaches every node, the graph is deemed fully connected; otherwise, the presence of disconnected components leads to the exclusion of its adjacency matrix from the set of feasible solutions.

Because a head and a tail must ultimately be connected to each other, a dummy atom is included in the set of groups as a placeholder and used to connect a head to a tail in problem P3. The dummy atom is represented by an asterisk (*) since it is treated by SMILES as a valid atomic symbol with an unspecified atomic number and a valence of 1. This allows the dummy atom to function like any other group in the valence-one set, occupying any available connection on other groups. Then, for both groups connected to the dummy atom in the head and tail structures, the bonds to the dummy atoms are replaced with a direct connection between them the groups, and therefore between the two substructures. The optimization was carried out using the default configuration of CPLEX solver. This includes tolerance levels, i.e., 10^–4^ for the relative MIP gap and 10^–6^ for the absolute MIP gap. The model is solved in Windows 10, Intel Core i5 −8365U CPU @ 1.60 GHz/1.90 GHz, RAM memory of 16,0 GB, 64-Bit operating system and x64-based processor. The computational time required to solve each structure is approximately 1.45 s.

### Additional Structural Molecular Constraints

The fundamental concept behind incorporating structural molecular constraints is to reduce the degrees of freedom within the structural model and to ensure “physically meaningful” structures. In addition to the connectivity constraints that arise from the valence of each group/atom, additional constraints can be incorporated to tailor the process of generating molecular structures to specific research requirements, for example to stay within a chemical space of interest where existing models of surfactant properties apply best. In fact, as suggested by He et al.^63^ in drug discovery, it may be of interest to maintain a constant part of the molecule (core), while substituting other parts (R groups) to increase the likelihood of achieving the desired properties. This concept can be applied when we want to ensure the integration of certain groups within each part of the surfactant, in which case these groups would be defined as the core of the head or tail, respectively. In this context, the core is defined as a single node, with the stipulation that all possible combinations must include it. Nevertheless, it is also particularly useful when studying surfactants that have a specific group (or groups) connected to the head. For instance, the presence of a benzene ring in the hydrophobic chain linked to the hydrophilic head is known to have the positive effect of decreasing the critical micelle concentration.^64^ Thus, it is possible to define a benzene ring attached to a sulfate group as the core of the molecule and designate the R group attached to the ring as the part of the structure that is subject to modification. In this case, the surfactant molecule is split into core and R-group, rather than purely head and tail, as illustrated in Figure 3.

**Figure 3:**
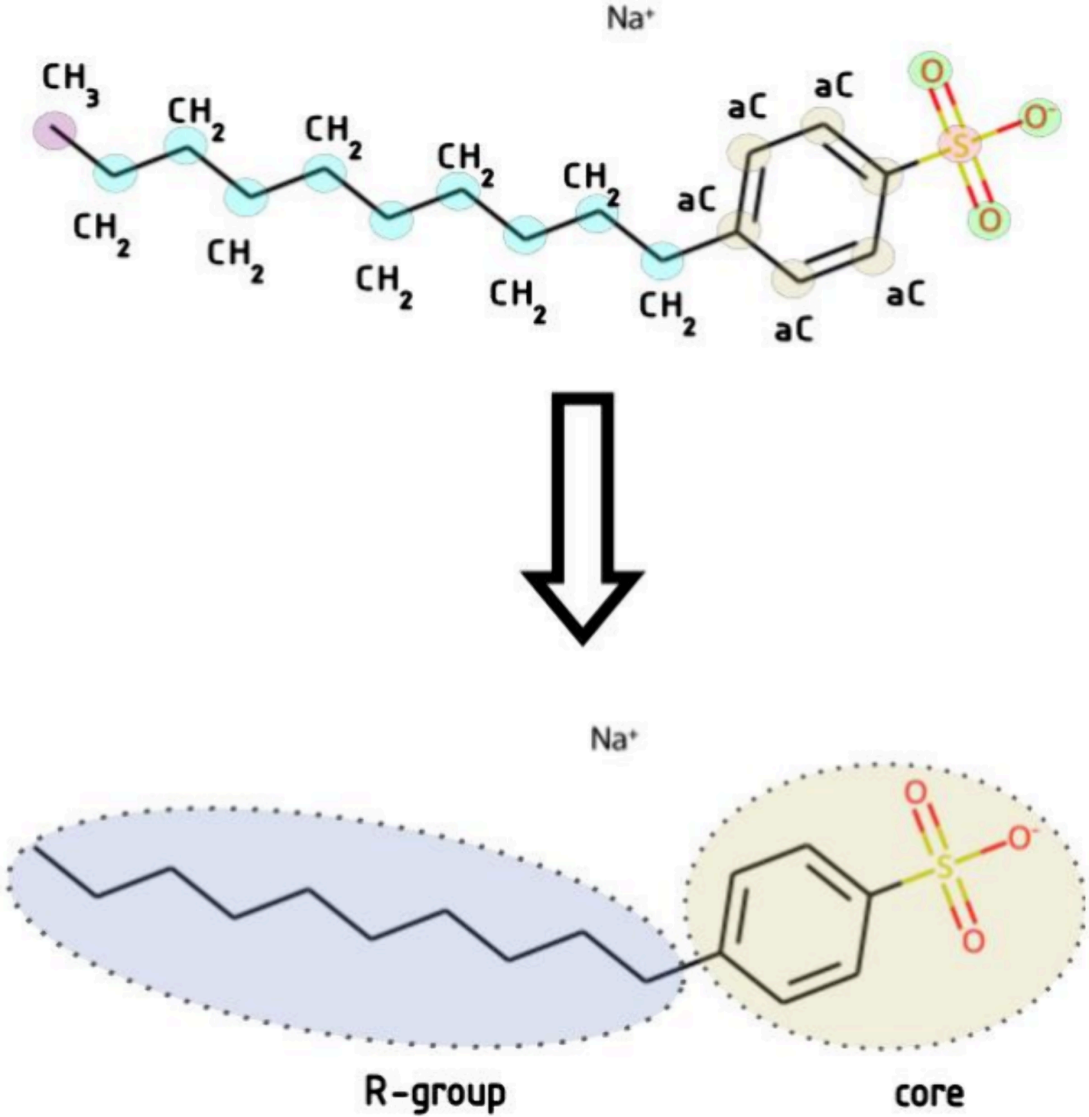
Core definition of molecule.

Other considerations that may be relevant in surfactant design can be included in this work, via restrictions in the generation of the bond matrices, allowing the position of bonds as well as bond types to be distinguished. First, it is possible to restrict which atoms of a molecular group can bind to another group, to avoid the formation of unwanted isomers. For example, we can distinguish the ortho, meta or para positions in an aromatic ring. As defined in Table 1, the disubstituted phenyl group has substituents in the 1 and 4 positions, which corresponds to a para configuration, eliminating ortho and meta configurations. Another constraint could be to establish which positions of an alkyl chain can have branching. For the disubstituted hexyl, connections in positions 1,3 and 4 are desired. Likewise, in the event of not wanting branching it is indicated that the bonding positions are only 1 and “final”, as occurs with heptylidene.

**Table 1:** Examples of Additional Restrictions Used in Bond Matrix Generation for Tails

group	SMILES	formula	group valence	valences for 1st connection (index 1)	2nd connection atom index	valences for 2nd connection	3rd connection atom index
disubstituted phenyl	C1=CC=CC=C1	C_6_H_4_	2	1	4	1	0
disubstituted hexyl	CCCCCC	C_6_H_11_	3	1	3	1	4
heptylidene	CCCCCCC	C_7_H_14_	2	1	final	1	0
methylidene	C	CH_2_	2	1	final	1	0

In SMILES notation, the explicit definition of hydrogen atoms is optional, being typically assumed to be implicitly considered. As a result, when a functional group is converted into its atomic adjacency matrix, hydrogen atoms do not appear as discrete entities. Instead, the number of implicit hydrogens for each atom is determined by the difference between its standard valence and the number of bonds it forms with other heavy atoms. Thus, we do not explicitly specify substituents in each group SMILE notation, allowing hydrogen atoms to remain implicitly handled. However, substituents are defined in the input data to ensure accurate molecular modifications by specifying the atom where a hydrogen bond is to be replaced, as shown in Table 1.

When linking two groups together, we do so by replacing implicit hydrogen atoms in both structures. Each group is first converted into its atomic adjacency matrix, where bonds are added at the specified connecting atom indices. Since hydrogens are not explicitly present in the matrix, they are automatically replaced as new bonds are introduced. Once the functional groups are connected within the complete head or tail bond atomic matrix, the structure is reconstructed and converted back into a SMILES representation. This approach ensures that hydrogen substitutions occur seamlessly while maintaining the correct valence of each atom.

By incorporating these constraints, according to the specific needs of each case, the space of feasible solutions is reduced, leading to a decrease in the computational time needed and the generation of a larger proportion of relevant molecular structures. The constraints described in this section are not meant to be exhaustive or mandatory for every application, but serve as illustrative examples of how the proposed framework can be flexibly adapted to different design goals by integrating chemically meaningful structural rules. The specific constraints used in each application are explicitly defined in the inputs for head and tail generation. Once the adjacency matrices have been generated, the heads and tails are represented as bond matrices.

### Surfactant Design

Having generated a set of tails and a set of heads, a Pareto-optimal set of surfactants is obtained by complete enumeration, computing the properties of all possible head/tail combinations. Furthermore, known surfactants of interest can also be added to the design space.

### Representation of Molecules

The surfactant molecules are generated by taking each (head, tail) combination, combining the corresponding bond matrices and replacing the two dummy atoms by a single bond between the tail atom bonded to a dummy atom and the head atom bonded to a dummy atom. The bond matrices corresponding to the complete surfactant molecules are then determined, and the RDKit python library is used to convert these matrices into simplified molecular-input line-entry specification (SMILES) notation.^65^ SMILES notation allows to generate a string that encapsulates information regarding atoms, bonds, rings, aromaticity, and branches. This string is obtained by following well-defined rules^66^ and serves as a concise expression of the molecule’s structure. To ensure the chemical validity of the generated structures, each molecule was sanitized using RDKit’s built-in routines. Molecules containing invalid atoms, valence violations, or disconnected fragments (i.e., those with a ″.″ in the SMILES string) were automatically identified and excluded. This filtering step was essential to prevent inconsistencies between the molecular graph and its corresponding SMILES representation. As a result, only topologically connected and chemically valid surfactant structures were retained. The overall success rate of the conversion process exceeded 98%, confirming the robustness and reliability of the approach. Additional information on how the head and tail matrices are combined to form the surfactant matrix, which is subsequently converted into SMILE notation, can be found in the Supporting Information and Github repository associated with this paper (see Supporting Information for further details).

### Surfactant Properties

The key properties considered in this work include the Synthetic Accessibility Score, CMC, surface tension, Krafft point, aquatic toxicity and biodegradability and are described in more detail in this section.

The Synthetic Accessibility Score (SAscore) evaluates the ease with which a molecule can be synthesized. It is measured on a scale from 1 (easy) to 10 (challenging).^67^ Voršilák et al.^68^ presented a score of 4.5 as the optimal threshold to distinguish between easy and hard-to-synthesize compounds. Therefore, a lower SAscore implies that the surfactant can be produced more practically and economically, making it a favorable choice for large-scale production.

The CMC is a vital property to evaluate during surfactant design. It refers to the concentration at which surfactant molecules form micelles in solution. Below the CMC, the surfactant molecules exist in solution as individual entities, while above it, they aggregate to form micelles. Moreover, the CMC is the point at which the surface tension of a solution reaches its lowest value.

The surface tension is also a critical property that impacts the behavior of surfactants at liquid–gas interfaces. Surfactants reduce surface tension by adsorbing at the liquid–air interface, thereby decreasing the attractive forces between liquid molecules. This reduction in surface tension allows the surfactant to spread more easily, enhancing wetting and spreading properties.

The Krafft Point is another important property to consider because it refers to the temperature at which an insoluble surfactant becomes soluble in water, forming micelles. Below the Krafft point, the surfactant molecules lack the thermal energy required to overcome hydrophobic interactions and form micelles. Designing surfactants with an appropriate (i.e., large enough) Krafft point ensures their stability and performance across a desired temperature range.^6^

From an environmental perspective, the biodegradability of surfactants is an important property to consider during design to reduce their impact on ecosystems and water bodies. A substance is considered readily biodegradable (RB) when it degrades by 60% in 28 days,^69^ so this property is assessed based on a metric that indicates the likelihood that a substance is readily biodegradable or not.^70^

Aquatic toxicity is also considered as water bodies are the most exposed to surfactant use. This is commonly quantified using EC50 or LC50 values.^71^ In this study, the LC50 is assessed, which represents the concentration of surfactant that causes the death of 50% of test organisms within a specific period of time,^72^ with high values of LC50 (and hence low toxicity) being desirable.^73^

### Property Prediction Models

Molecular descriptors have been widely used as inputs for data-driven models to predict molecular properties, using linear regression, decision trees, support vector machines and different types of neural networks.^74^ A molecular descriptor is defined as the “final result of a logical and mathematical procedure, which transforms chemical information encoded within a symbolic representation of a molecule into a useful number or the result of some standardized experiment”.^75^ In this work, predictive models of the properties used in the optimization problems are developed using Mordred descriptors^76^ as input. Given a database of molecules for which data on a property of interest are available, the Mordred descriptors are obtained for each molecule via a highly flexible Python package that generates 1,826 descriptors per molecule, based on the SMILES string. The types of molecular descriptors available in Mordred include 0D descriptors (e.g., count of different atom types), 1D descriptors (e.g., count of different functional groups), 2D descriptors (e.g., based on atom connectivity) and 3D descriptors (e.g., atom distances at an optimized geometry). This results in a large number of descriptors, which must be reduced to generate statistically significant and tractable models. A first filtering is thus performed by eliminating descriptors for which some values are missing, are non-numerical, or are identical for all molecules in the database. Then, the number of descriptors is further reduced by calculating the Pearson correlation coefficient for each pair of descriptors, based on all descriptor values obtained for all molecules. To avoid redundancy, descriptors showing a high absolute correlation (coefficient >0.9, based on typical thresholds found in the literature^77^) with more than two other features were removed. This is because highly correlated features tend to provide overlapping information, which may introduce bias or instability in the model. In the case of descriptors involved in only one highly correlated pair, the second descriptor is discarded from the set of descriptors. The output of this descriptor filtering process is illustrated in Figure 4 for the database of 269 surfactant molecules used to build a model of the synthetic accessibility score. Each element in the matrix represents the linear correlation between two descriptors, with values ranging from 0 (no correlation) to 1 (perfect correlation), as indicated by the color scale. Darker diagonal elements correspond to perfect self-correlation, while the sparse and low-intensity off-diagonal values reflect the low extent of correlation among the selected descriptors. The approach results in the reduction of the number of descriptors to 221. Once the number of descriptors has been reduced, the database is split randomly into 80% training and 20% testing data. Scikit-learn is then used to investigate different modeling approaches, using functionality for data transformation, supervised learning, and model evaluation and selection.^78^ The mathematical form of each property model is chosen to achieve good balance between accuracy and statistical significance, with as simple a mathematical form as possible. Different supervised learning approaches are tested for each property: linear models derived with Lasso regression or Partial Least Square (PLS) regression as well as artificial neural network (ANN) models. The selection of the final model for each property is based on the evaluation of standard performance metrics, specifically the coefficient of determination (R^2^) and the root-mean-square error (RMSE), computed on both training and test sets.

**Figure 4:**
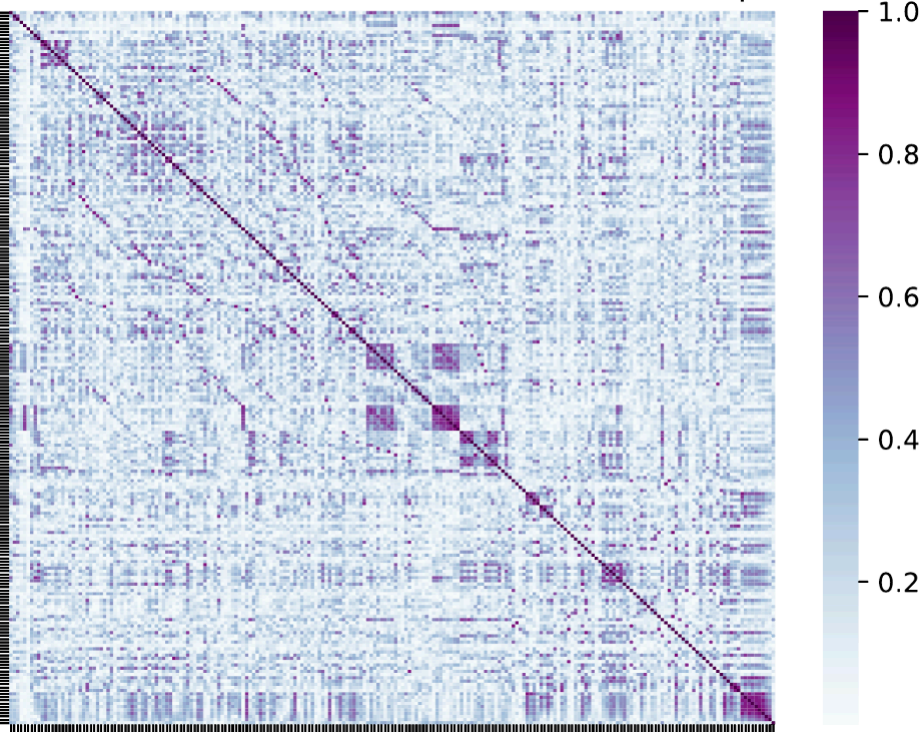
Schematic representation of the matrix of Pearson correlation coefficients for 221 Mordred descriptors, obtained from a starting set of 1826 descriptors. Each descriptor is characterized by a vector of 269 values, derived from a database of 269 surfactant molecules. The value of each correlation coefficient is indicated by the color scale and highlights the low extent of correlation between the final descriptors.

### Multiobjective Optimization: Surfactant Design

Once the models for the prediction of all selected properties have been developed, they can be applied effectively to the generated head and tail combinations to identify those that meet the specific requirements of each surfactant design task. In this work, a multiobjective optimization is required, since we need to balance at least one key physicochemical surfactant property with at least one environmental metric. However, in Multi-Objective Mathematical Programming (MMP), there is no single optimal solution that can optimize all the objectives simultaneously. In such cases, decision makers seek the ″most preferred″ solutions rather than the optimal one. In MMP, the traditional notion of optimality is replaced by the concept of Pareto optimality or efficient solutions, which are those that cannot be enhanced in one objective function without compromising their performance in at least one of the remaining objectives.^79^ Recent advances in MMP have found increasing relevance in CAMD problems, particularly in areas such as solvent^80^ and working fluid design,^81^ as well as drug discovery.^82^ The most used multiobjective optimization methods include scalarization approaches, such as the weighted sum (WS) and sandwich (SD) algorithms, metaheuristic-based strategies such as Nondominated Sorting Genetic Algorithm II (NSGA-II), and the widely used epsilon-constraint technique. CAMD problems are characterized by a high degree of nonlinearity/nonconvexity and recent progress has involved incorporating global search techniques, such as Multi-Level Single Linkage (MLSL), into scalarization methods with the aim of improving the capacity to identify globally optimal approximations of the Pareto front.^83^ A known limitation of WS and SD methods is that they can only identify solutions in the convex regions of the Pareto front.^84^ This issue can be effectively addressed by using epsilon-constraint methods although generating well-distributed points on the Pareto front can be challenging. The ε-constraint method, a practical and straightforward approach, is used to transform the MMP problem into a single objective optimization subproblem, which can be solved repeatedly for different values of the ε constant(s), resulting in a Pareto set of solutions. Thus, one of the objective functions is optimized (here, by enumeration) using the other objective functions as constraints. If there are more than two objectives, a separate constraint, with a different ε value, is included for each additional objective.^85^ Considering that all objective functions are to be maximized, the problem is formulated as follows, where S corresponds to the feasible solution space:

$$\underset{y\in S}{\max}f_{1}(y)\;\mathrm{Subject}\;\mathrm{to}\;f_{2}\left(y\right)\geq \varepsilon_{2},f_{3}\left(y\right)\geq \varepsilon_{3},\cdot \cdot \cdot ,f_{p}\left(y\right)\geq \varepsilon_{p}$$ 12

For minimization, each objective function expressed as a constraint would be set to be less than or equal to the ε parameters.

To implement this method, it is necessary to determine the range of at least *p* – 1 objective functions to define appropriate ranges of values for the ε parameters (ε_2_, ···, ε*_p_*). The range *r*_*i*_ of each objective function *f*_*i*_ is determined as follows:

$$r_{i}=f_{i}^{\max}-f_{i}^{\min},i=2,\cdot \cdot \cdot ,p$$ 13

Then, *r*_*i*_ is divided into *q*_*i*_ equal intervals using (*q*_*i*_ −1) intermediate equidistant grid points. Thus, we have (*q*_2_ + 1), ···, (*q*_*p*_ + 1) grid points for *f*_2_, ···, *f*_*p*_,respectively. The number of subproblems to solve becomes Π_*i* = 2_^*p*^(*q*_*i*_ + 1). The following values of ε_*i*_^*k*^are then used to formulate the ε constraints, where *k* is the grid point number:

$$\varepsilon_{i}^{k}=f_{i}^{\max}-k\cdot \frac{r_{i}}{q_{i}},k=0,\cdot \cdot \cdot ,q_{i};i=2,\cdot \cdot \cdot ,p$$ 14

Increasing the number of grid points results in a denser approximation of the Pareto front but at the expense of higher computational costs. Therefore, a trade-off between the granularity of the efficient set and computational effort is always recommended.^79,86^

### Implementation

The Python programming language has gained significant prominence in scientific computing, primarily due to its remarkable efficiency, making it an ideal choice for the implementation of the proposed formulation. Moreover, Python’s strength lies in its extensive collection of high-quality numerical libraries like NUMPY or SCIPY, and specialized ones like RDKit, which deal with molecular design tasks. These libraries empower researchers to not only tackle complex problems efficiently but also facilitate molecular structure visualization and graphical representation, among various other capabilities. The Python Optimization Modeling Objects (Pyomo) software package is used for modeling and optimization.

P1 and P2 (Figure 1) are implemented in separate python scripts that read the inputs (molecular groups and specific restrictions desired in each structure) from an Excel file, with the format shown in the case studies. Solutions are stored in pickle files (.pkl extension) as adjacency matrices and SMILES notation. The script for P3 includes molecule generation, property evaluation and the solution of the multiobjective optimization problem. The heads and tails are imported from pickle files and each property prediction model developed is imported from two different files, with the extensions.joblib and.joblibparameters. The.joblib files contains the model itself, while the.joblibparameters contains the list of descriptors needed to use the model. The rest of the input data needed for optimization (multiple objective functions, physical property constraints, and real surfactants from external databases, if applicable) are entered from the Excel file and the code with models needed for reproducing our results as well as the generated structures are available in the GitHub repository.

## Results and Discussion

### Property Prediction Models

The performance of the predictive models developed to compute the relevant properties as a function of Mordred molecular descriptors is summarized here.

Synthetic Accessibility Score (SAscore): A database containing 269 surfactants is constructed from the model developed by Ertl and Schuffenhauer.^67^ Descriptor filtering reveals that 221 Mordred descriptors are required (see Figure 4). Partial least-squares regression (PLS regression) is applied with 4 principal components (PCs).
log­(CMC): the model to predict critical micelle concentration is built with data for 353 surfactants, combining data published by Qin et al.,^5^ Rosen & Kunjappu^20^ and Seddon et al.^6,21,22^ The number of descriptors decreases to 223 after filtering. PLS regression is applied with 7 PCs.
Surface tension: A PLS regression model is built to predict surface tension, using 5 PCs constructed from 130 descriptors using data published by Seddon et al.^21^ for 91 surfactants. It should be noted that their model is available for direct use. Their approach separately predicts the Langmuir constant, CMC, and maximum surface excess concentration using different sets of molecular descriptors, which are subsequently substituted into the Szyszkowski equation^87^ to estimate surface tension. However, to ensure mathematical simplicity and consistency across all proposed models, we have developed our own multiple linear model following the same methodology as for the other properties.
Krafft point: a model to predict the Krafft point is built with available data published by Negin et al.^88^ for 45 surfactants. The number of descriptors decreases from 1,826 to 67 after filtering. Lasso regression is used with 5-fold cross validation to build a multiple linear model, decreasing the number of nonzero parameters (i.e., descriptors) to 22.
log­(LC50): Data on the baseline toxicity of neutral organic chemicals for fish, namely 96 h LC50 data, are gathered for 267 molecules, from ECOSAR, available in the Estimations Programs Interface for Windows.^73^ The number of descriptors decreases to 217 after filtering. Lasso regression with 5-fold cross validation is used to construct a multiple linear model with 68 nonzero parameters, based on the logarithm of LC50 values.
Biodegradability: Data for 267 surfactants is obtained from US EPA,^73^ within which the standalone program BIOWIN (v4.11) is usedewith 5-fold cross validation is used to construct a multiple linear model with 28 nonzero parameters.

The CMC and LC50 models are built with data converted into logarithmic scale. This transformation is common in QSPR data processing as property values can vary by several orders of magnitude for different molecules. The performance metrics for all models are reported in Table 2 and parity plots shown in Figure 5. Overall, the models demonstrate adequate predictive capabilities. However, the surface tension and Krafft point models exhibit weaker test performance in terms of R^2^ and RMSE, respectively. This is likely due to the limited data set size, which constrains the models’ ability to capture variability, ultimately reducing their generalization capacity.

**Table 2:** Metrics for the Property Prediction Models^a^

model	units	size of data set	model type	**RMSE_train_**	***R*_train_^2^**	**RMSE_test_**	***R*_test_^2^**
SAscore		269	PLS	0.24	0.90	0.21	0.91
log(CMC [μM])		353	PLS	0.47	0.86	0.47	0.87
surface tension	mN/m	91	PLS	1.79	0.92	3.24	0.68
Krafft point	°C	45	LassoCV	1.70	0.99	4.44	0.93
log(LC50 [mg/L])		267	LassoCV	0.58	0.81	0.62	0.80
Biodegradability		267	LassoCV	0.13	0.86	0.11	0.86

a The size of the dataset denotes the total number of training and testing data. RMSE_*x*_ denotes the root mean squared error for the training (*x* = train) and testing (*x* = test) sets and carries the same units as the property of interest. Similarly, *R*_*x*_^2^ denotes the coefficient of determination for the training and testing sets, and it is dimensionless.

**Figure 5:**
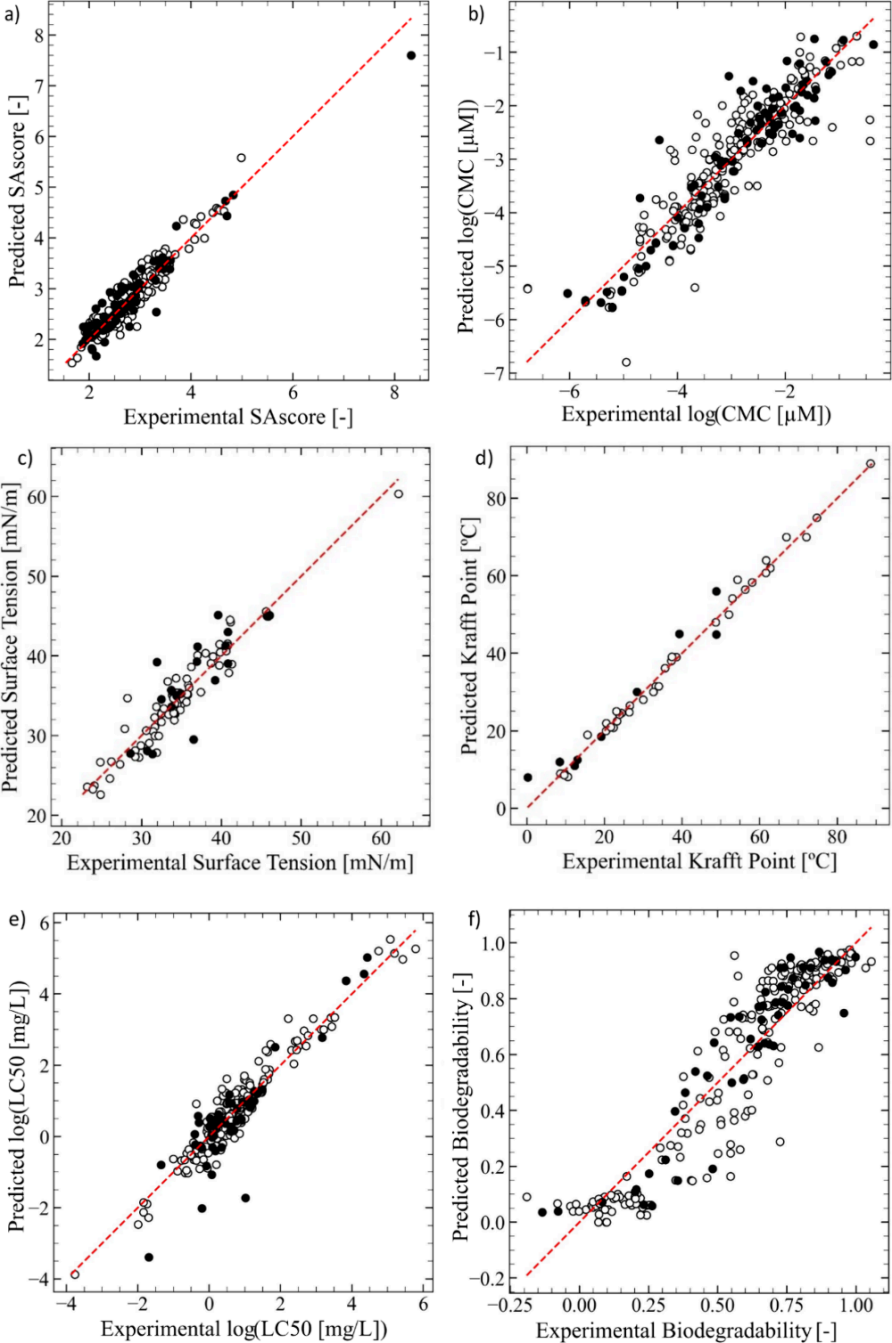
Parity plots showing the predicted values of the relevant properties vs the corresponding experimental values for (a) synthetic accessibility score, (b) log­(CMC), where CMC is in μM, (c) surface tension, (d) Krafft point, (e) log­(LC50), where LC50 is in mg/L and (f) biodegradability predictive models. The empty circles represent the training set, and the filled circles represent the test set.

### Case Studies

Two illustrative case studies are presented with different multiobjective optimization formulations. The predictive property models described above are used. Head and tail groups in Tables 3 and 4 are selected based on the most frequent functional groups found in the data sets used to train the predictive models to ensure the models are used within their applicability domains and that industrially relevant molecules are more likely to be designed.

**Table 3:** Head Inputs: Definition of Groups, Valences, and Other Constraints^a^

groups	SMILES	group valence	valence first connection atom	second connection atom index	valence second connection atom	maximum number of groups
dummy	*	1	1	0	0	1
sulfate	OS(=O)(=O)[O−]	1	1	0	0	1
sulfinate	O=S(=O)[O−]	1	0	2	1	1
ketone	C=O	2	2	0	0	1
ester	C(=O)O	2	1	final	1	1
*N*,*N*-dimethylaminomethylene	CN(C)C	1	1		0	1
glycoside	C([C@@H]1[C@H]([C@@H]([C@H](C(O1)O)O)O)O)O	1	1	0	0	1
methylidene	C	2	2	0	0	2
methyl	C	1	1	0	0	1
methyllidyne	C	3	3	0	0	1
amide	C(=O)N	2	1	final	1	1
dimethylammonium oxide	[N+](C)(C)[O-]	1	1	0	0	1
ethoxy	CCO	2	1	final	1	1
hydroxyl	O	1	1	0	0	1
phosphate	OP(=O)(O)O	1	1	0	0	1

a In the event that more atom connections need to be specified, supplementary columns may be added.

**Table 4:** Tail Inputs: Definition of Groups, Valences, and Other Constraints^a^

group	SMILES	group valence	valence first connection atom	second connection atom index	vlence second connection atom	maximum number of groups
dummy	*	1	1	0	0	1
methylidene	C	2	1	final	1	1
methyl	C	1	1	0	0	3
methyllidyne	C	3	3	0	0	3
vinylene	C=C	2	1	final	1	1
alkylene	C#C	2	1	final	1	1
hexylene	CCCCCC	2	1	final	1	1
disubstituted phenyl	C1=CC=CC=C1	2	1	4	1	1

a In the event that more atom connections need to be specified, supplementary columns may be added.

To generate the surfactant heads, 14 chemical groups, in addition to the dummy atom, are chosen as building blocks, as shown in Table 3. All groups can appear only once in a givenhead except for the CH_2_ group, which can appear up to twice (*n*_*CH*_2__ = 2).

Furthermore, the powerset φ(J) , which refers to the set of all possible combinations of groups belonging to the set of groups J as defined in eq 6 of section 3.1.3, is limited to combinations with a minimum of 2 groups and a maximum of 4 groups.

The first problem P1, generation of possible heads, is solved and any repeated solutions are eliminated. As a result, 102 heads are generated and sent to the third optimization problem P3 instead of the 16,384 (2^14^) possible combinations that would have been obtained without the added constraints. The molecular structures and the corresponding SMILES notation for the generated heads are provided in the Supporting Information.

To generate the tails through the second problem P2, 7 different groups in addition to the dummy atom are defined as shown in Table 4. As disubstituted phenyl is defined as a chemical group, the connecting atoms within the aromatic rings are specified (e.g, the para position, where second connection atom has an index of 4). Note that atom indexes correspond to the order of the atoms in the SMILES notation. Some groups can appear more than once: the CH_3_ group, with up to 3 instances, and the CH group, with up to 3 instances. The powerset is limited to combinations with a minimum of 3 groups and a maximum of 7 groups, always including hexylene as one of them in order to ensure a minimum linear chain length.

The feasible solutions to problem P2 leads to 50 possible tails. The molecular structures and the corresponding SMILES notation for the generated tails are provided in the Supporting Information. This leads to the formation of 5100 candidate surfactants (102 heads x 50 tails). Additionally, 270 widely used surfactants that cover another part of the molecular space in terms of the groups they contain are added from an internal P&G database. The third problem P3 can then be solved. For both illustrative case studies, the range of each objective function is divided into 10 intervals, resulting in 11 grid points, consistent with values commonly reported in the literature.^79,89–91^ However, the proposed multiobjective framework is flexible and the value of each *q*_*i*_ can be adapted to the problem complexity and computational budget.

In the first case study, a multiobjective optimization of the following surfactant properties is carried out: maximization of biodegradability and minimization of CMC and synthetic accesibility score, with constraints on the values of the surface tension, Krafft point and the toxicity as described in Table 5. The resulting Pareto front is shown in Figure 6.

**Table 5:** Model Inputs for the Final Step of Surfactant Optimization in the First Case Study: Definition of Objecitve Functions and Cut-Off Values for Constraints

model	objective function/constraint	max/min	min	max	unit
SAscore	objective function	min			
Krafft point	constraint		0	inf	°C
surface tension	constraint		20	40	mN/m
log(CMC [μM])	objective function	min			
log(LC50 [mg/L])	constraint		0	inf	
biodegradability	objective function	max			

**Figure 6:**
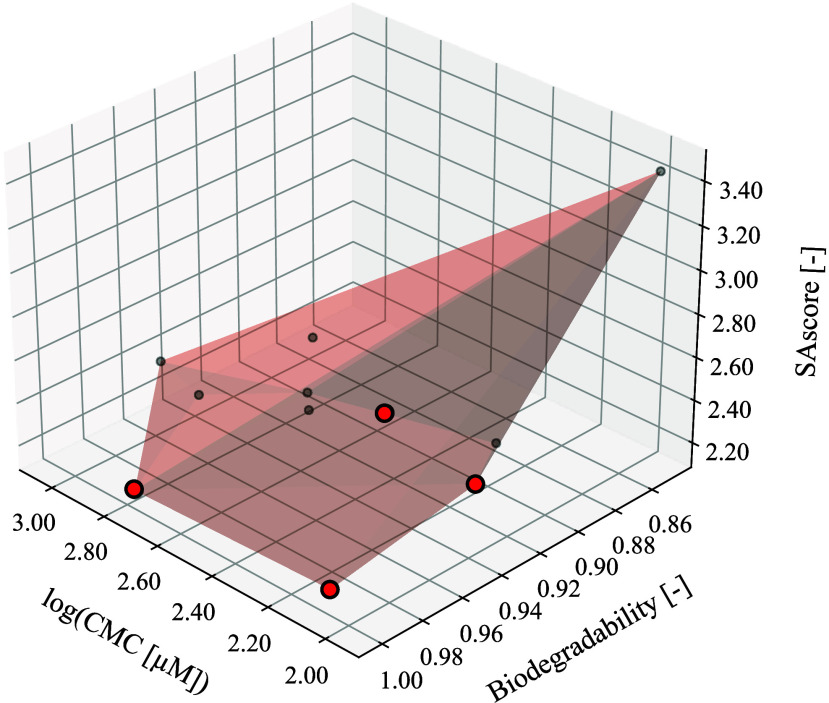
Pareto front for the first case study, where both SAscore and log­(CMC), where CMC is in mg/L, are minimized and biodegradability maximized. The red points represent the optimal solutions.

The selection of optimal molecules from the Pareto front is performed using the Technique for Order Preference by Similarity to Ideal Solution (TOPSIS). TOPSIS evaluates each candidate solution by calculating its Euclidean distances to both the ideal (best) and negative-ideal (worst) points, which are defined based on the normalized objective values.^92^ In this work, objective values are normalized using the maximum-minimum method prior to distance calculations. A compromise score, known as the closeness coefficient, is then computed for each solution to quantify its relative proximity to the ideal solution while maximizing the distance from the negative-ideal solution. This approach enables a systematic and balanced evaluation of trade-offs among multiple conflicting objectives, as illustrated by the red circles in Figure 6. Given the early stage nature of this design study, four solutions were selected from the Pareto front to facilitate potential experimental validation of different trade-offs. The best four, as depicted in Figure 7, have all been generated from the solution of problems P1 and P2 and do not correspond to any of the molecules added from the external database. The predicted property values for each of these molecules are shown in Table 6.

**Figure 7:**
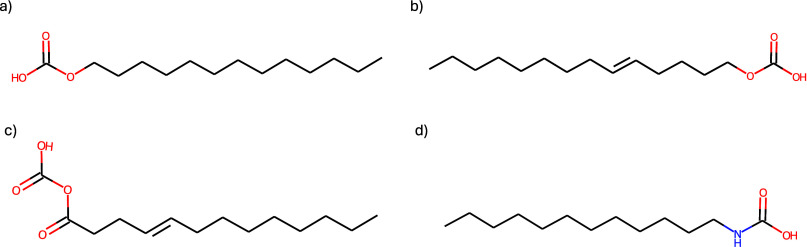
Optimal solutions of the multiobjective optimization problem in the first case study. Molecules from (a) to (d) are presented in order of increasing predicted CMC values. The corresponding SMILES are (a) CCCCCCCCCCCCCOC­(=O)­O, (b) CCCCCCCCC=CCCCCOC­(=O)­O, (c) CCCCCCCCC=CCCC­(=O)­OC­(=O)­O, and (d) CCCCCCCCCCCCNC­(=O)­O.

**Table 6:** Predicted Values of Properties for the Selected Optimal Molecules Shown in Figure 7 for the First Case Study

molecule	SAscore (−)	Krafft point (°C)	log(LC50 [mg/L])	biodegradability (−)	surface tension (mN/m)	log(CMC [μM])
a	2.21	4.43	0.30	1.00	27.8	2.06
b	2.33	1.11	0.66	0.90	28.4	2.16
c	2.61	4.93	0.81	0.95	26.7	2.25
d	2.18	3.37	0.54	1.00	26.5	2.77

A second case study is proposed with the same candidate structures but changing the aim of the multiobjective optimization to the maximization of the LC50 value (i.e., minimization of toxicity) and minimization of the synthetic accesibility score, restricting the values of the other properties as shown in Table 7.

**Table 7:** Model Inputs for Final Step of Surfactant Optimization for the Second Case Study: Definition of Objective Functions and Cut-Off Values for Constraints

model	objective function/constraint	max/min	min	max	units
SAscore	constraint		0	4.5	
Krafft point	constraint		0	inf	°C
surface tension	constraint		20	35	mN/m
log(CMC [μM])	objective function	min			
log(LC50 [mg/L])	objective function	max			
biodegradability	constraint		0.6	1	

The resulting two-dimensional Pareto is shown in Figure 8. The distribution of toxicity values is very heterogeneous, in line with the database used to build the predictive model. Once again, based on the normalized Euclidean distances, the solutions exhibiting the lowest compromise scores are denoted in red, signifying that they constitute the optimal molecules that effectively balance the trade-offs among the two conflicting objectives.

**Figure 8:**
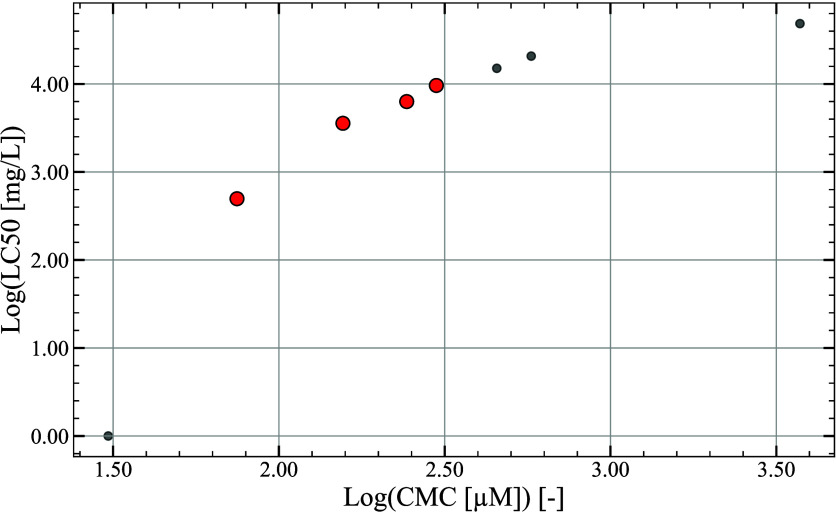
Pareto front for the second case study based on log­(CMC) (minimized), where CMC is in μM, and log­(LC50), where LC50 is in mg/L, (maximized). The red points represent the best solutions.

The molecules selected as the best are shown in Figure 9, and they have been newly generated from the combination of designed heads and tails. As can be noted, all four molecules include the glucose group. This is encouraging given that a limited number of biosurfactants that also include glucose are currently being manufactured at industrial scale, such as alkylpolyglucosides (APG), which are nonionic surfactants derived from sustainable sources like fatty alcohols and sugars. In comparison to their chemical counterparts, it has been demonstrated that these compounds offer distinct advantages, including reduced toxicity and enhanced biodegradability.^93^ The predicted property values for each of the selected molecules are shown in Table 8.

**Figure 9:**
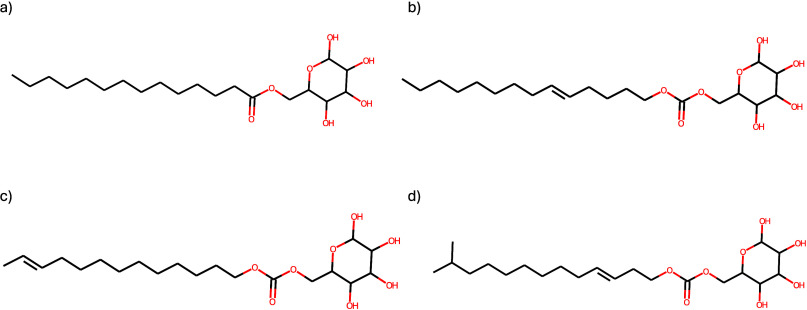
Optimal solutions of the multiobjective optimization problem for the second case study. Molecules (a)–(d) are presented in order of increasing predicted CMC values. The corresponding SMILES are (a) CCCCCCCCCCCCCC­(=O)­OCC1OC­(O)­C­(O)­C­(O)­C1O, (b) CCCCCCCCC=CCCCCOC­(=O)­OCC1OC­(O)­C­(O)­C­(O)­C1O, (c) CC=CCCCCCCCCCCOC­(=O)­OCC1OC­(O)­C­(O)­C­(O)­C1O, and (d) CC­(C)­CCCCCCCC=CCCOC­(=O)­OCC1OC­(O)­C­(O)­C­(O)­C1O.

**Table 8:** Predicted Values of the Properties of the Selected Optimal Molecules in the Second Case Study as Shown in Figure 9

molecule	SAscore (−)	Krafft point (°C)	log(LC50 [mg/L])	biodegradability (−)	surface tension (mN/m)	log(CMC [μM])
a	3.39	77.30	2.70	0.76	33.2	1.87
b	3.55	91.00	3.55	0.74	32.1	2.19
c	3.57	87.43	3.80	0.77	31.8	2.39
d	3.76	99.58	4.00	0.72	31.1	2.48

Interestingly, some of the designed molecules have been previously reported in the literature, showing experimental properties that are consistent with our predictions supporting the reliability of the proposed models.^94^ The proposed molecules ensure effective biodegradability and reduced toxicity without compromising the technical performance properties when compared to some existing surfactants in the market, such as Sodium dodecyl sulfate (SDS), Didodecyldimethylammonium bromide (DDAB), dodecyl dimethyl phosphine oxide (C12DMPO), and Dodecyldimethylamine N-oxide (DDAO). As outlined in Table 9, both SDS and DDAO demonstrate relatively high biodegradability and lower toxicity when compared to C12DMPO and DDAB.^75–82^ In comparison, the SAscore is the only property that may be slightly jeopardized in the proposed less toxic molecules from the second case study, although it still remains well below the previously established threshold of 4.5. Meanwhile, the molecules from the first case study demonstrate significantly better biodegradability while maintaining comparable or even better SAscore, CMC, and LC50 values.

**Table 9:** Literature and Predicted Values (Using Our Models) for the Same Properties that Serve as Objective Functions in the Presented Case Studies of Common Surfactants, Provided for Comparison

	measured values				predicted values			
surfactant	**SAscore (−)** ^67^	**log(CMC [μM])**	**Biodegradability (−)** ^73^	**log (LC50**[mg/L])^73^	**SAscore (−)**	**log(CMC [μM])**	**biodegradability (−)**	**log(LC50** [mg/L])
DDAB	2.05	2.24 ^22^	0.17	–2.00	2.24	1.60	0.25	–1.30
DDAO	2.36	3.20 ^95^	0.87	0.62	2.23	3.13	0.85	0.82
SDS	2.63	3.91 ^22^	0.95	0.82	2.71	4.20	0.96	0.86
C12DMPO	2.32	2.48 ^22^	0.63	–0.70	2.51	1.70	0.81	–0.40

In general, the models show good agreement with the literature data and perform especially well for SAscore with a mean relative error of 6.5%. For log­(CMC), the average relative error is about 17.4%, which is within the range of experimental uncertainty typically associated with CMC measurements due to variations in ionic strength, temperature or measurement technique.^96^ As expected, biodegradability and log­(LC_50_) predictions exhibit the highest errors among the modeled properties, achieving relative errors of 19.7 and 28.8%, respectively. As highlighted by Hrovat et al.^97^ in their study on the variability of toxicity data within databases, LC_50_ values often span several orders of magnitude due to inconsistencies in experimental conditions, incomplete reporting (e.g., missing life stage, pH, temperature, or water hardness), and biological variability across species and developmental stages. These factors introduce substantial uncertainty, complicating the development of more accurate predictive models. Nonetheless, the predictions remain within acceptable margins for early stage design.

Overall, the results illustrate the ability of the new CAMD framework to identify potential sustainable surfactant molecules that can be used in consumer products depending on the desired properties of the products.

## Conclusions

The proposed framework enables the design of sustainable surfactant molecules, integrating the optimization of crucial properties related to technical performance while considering sustainability, via biodegradability and toxicity metrics. The generation of candidate constituent parts, namely hydrophilic and hydrophobic segments, from a set of groups, to make up a surfactant molecule, is carried out first with the inclusion of structural constraints that can be chosen by the designer. The generated constituent parts are then combined to obtain candidate surfactants. These, as well as molecules from databases, can then be assessed via property-prediction techniques developed based on available data, deploying techniques such artificial neural networks, partial least-squares or multilinear regression models, as appropriate. The molecules are ranked using constrained multiobjective optimization to generate a set of Pareto-optimal solutions. This approach facilitates an initial screening out of molecules that may not be suitable for the specific case under investigation. Furthermore, it streamlines the exploration of the chemical space to be studied, resulting in the rapid and efficient identification of promising surfactant molecules.

The approach has been applied to two case studies, placing particular emphasis on the realm of detergents due to their prevalent application. The approach was found to be effective in the search of biodegradable and more environmentally friendly surfactants, while mantaining good technical performance. With a set of 21 atom groups and selected constraints, 102 heads and 50 tails were generated out of 2^21^ possible structures that would be obtained without constraints. This results in over 5,000 molecules to be screened. Additionally, the framework enables swift comparison of the predicted properties of the designed compounds with the properties of known surfactants of interest. The candidate surfactants include promising sugar-based surfactants with excellent biodegradability and toxicity profiles.

The approach has been described and tested for the generation of “traditional” surfactants consisting of a single head and a single tail, but the proposed workflow can easily be adapted to the generation of other interesting structures. Future extensions of the framework could include its adaptation to Gemini surfactants, which would require a redefinition of headgroup inputs and the introduction of structural constraints to ensure the presence of two head structures per molecule. Concurrently, we are developing an extension for multicomponent surfactant systems, where accurate modeling of intersurfactant interactions, strongly dependent on the nature of each component, is essential to accurately capture mixture behavior. The framework can also be extended to include the consideration of other relevant properties or their dependence on conditions such as temperature, as well as additional constraints related to chemical stability that would enhance the framework by preventing the generation of unstable or undesired structures. In parallel, limited data availability for certain properties may affect model reliability and generalizability. Future work should prioritize expanding experimental data sets to address this limitation. This study is limited by the exclusive use of 2D molecular descriptors, which may constrain predictive accuracy for properties influenced by molecular conformation. 2D descriptors offer a significant advantage in terms of computational efficiency and are widely used as a first step in screening pipelines in both academic and industrial settings. Nevertheless, incorporating 3D structural information could further improve model accuracy, particularly for interfacial properties, and can be considered in future developments of the framework. Finally, from an industrial perspective, challenges remain in translating computationally designed surfactants into viable products. These include the rapid experimental assessment of the feasibility of synthetic routes, availability of starting materials, production costs, and scalability. Addressing such factors will be critical to bridge the gap between in silico design and practical implementation.

## Supplementary Material



## Data Availability

Supplementary data and associated models related to this article can be found on the GitHub repository https://github.com/SofiaGNu/Design-of-Surfactant-Molecules-Under-Performance-Constraints and are available under a CC BY 4.0 license.

## Notes

Custom Python functions for constructing complete surfactant molecules from head and tail matrices and converting them to SMILES notation; bond matrices for head, tail, and complete molecule example (Tables S1–S3, Figure S1); molecular structures of 102 generated head fragments (Figures S2–S103) and 50 generated tail fragments (Figures S104–S153) (PDF)
