# Supplementary material for: Design of Surfactant Molecules Under Performance Constraints
Source: ACS Sustain Chem Eng. 2025 Aug 20;13(34):13808–24. doi: 10.1021/acssuschemeng.5c04112 (PMC12406250; doi:10.1021/acssuschemeng.5c04112)
Supplement: Supplementary file 1 [file sc5c04112_si_001.pdf]

Supporting Information

Design of Surfactant Molecules Under Performance Constraints

Sofía González-Núñez[a], Zeynep Sumer[b], Carlos Amador[c], Prakash Madhav[d], Ajay Muralidharan[d], Claire S. Adjiman[b], Mariano Martín[a1]

[a] Departamento de Ingeniería Química. Universidad de Salamanca. Pza. Caídos 1-5, 37008 Salamanca, Spain
[b] Department of Chemical Engineering, Sargent Centre for Process Systems Engineering, Institute for Molecular Science and Engineering, Imperial College London, South Kensington Campus, London SW7 2AZ, United Kingdom
[c] Newcastle Innovative Centre. Procter and Gamble. Whitley Rd, Longbenton, Newcastle Upon Tyne, Tyne And Wear NE12 9SR, England
[d] Mason Business and Innovation Center. Procter and Gamble. Mason-Montgomery Rd, Mason, OH 45040, United States

Number of Pages: 59

Number of Tables: 3

Number of Figures: 153

[1] M. Martín mariano.m3@usal.es

Table of Contents

FROM MATRIX TO SMILE NOTATION……………………………………………………………………………S3

GENERATED HEADS…………………………………………………………………………………………..S9

GENERATED TAILS…………………………………………………………………………………………..S43

FROM MATRIX TO SMILE NOTATION

The entire process of combining the Head and tail matrices into a complete surfactant molecule, as well as its subsequent conversion into SMILES notation, is performed by the custom function *"combination_head_tail"*. This function relies on an additional user-defined function, "*MolfromGraphs"*, which constructs an RDKit molecular object from the adjacency matrix and atom list representation of the surfactant. These functions require the external libraries pandas and RDKit, specifically the Chem module from RDKit.

The function "*combination_head_tail"* combines the adjacency matrices of the surfactant's Head and tail segments into a single adjacency matrix representing the entire molecule. It aligns and merges the two matrices by inserting zero-filled rows and columns to ensure consistent indexing, then connects the Head and tail by forming a bond between designated attachment points. After constructing the full adjacency matrix, the function extracts atomic information, uses the auxiliary function "*MolfromGraphs"* to generate an RDKit molecular object, and finally converts it into SMILES notation. The function returns the adjacency matrix, the SMILES string, and the RDKit molecule object representing the complete surfactant molecule.

```
def combination_head_tail(head,tail):
  data_df_new=copy.copy(tail)
  data_df_new.rename(columns={"*": "*_"}, index={"*": "*_"}, inplace=True)
  data_df_head=copy.copy(head)
  z=data_df_new.columns.get_loc("*_")
  Number_of_columns_to_insert_to_tail=len(data_df_head)
  if Number_of_columns_to_insert_to_tail == 0:
            d=0
  else:
    d = pd.DataFrame(np.zeros((Number_of_columns_to_insert_to_tail, 0)))
    d=d.set_index(data_df_head.columns)
    data_df_new = pd.concat([data_df_new.iloc[:z], d, data_df_new.iloc[z:]])
```

```
data_df_new.fillna(0, inplace=True)
Number_of_rows_to_insert_to_head=(len(data_df_new))-(len(data_df_head))
e=pd.DataFrame(np.zeros((Number_of_rows_to_insert_to_head, 0)))
data_df_head.loc["del"]=0
w=data_df_head.index.get_loc("del")
data_df_head = pd.concat([data_df_head.iloc[:w], e, data_df_head.iloc[w:]])
data_df_head.fillna(0, inplace=True)
data_df_head=data_df_head.drop(("del"), axis=0)
data_df_head=data_df_head.set_axis(data_df_new.index.tolist(),axis=0)
surfactant = pd.concat([data_df_head,data_df_new], axis=1)
old_index=surfactant.index.tolist()
surfactant=surfactant.reset_index()
index = surfactant.index
surfactant=surfactant.drop(("index"), axis=1)
condition = surfactant["*"]==1
loc1 = list(index[condition])
index = surfactant.index
condition = surfactant["*_"]==1
loc2 = list(index[condition])

surfactant.iloc[loc1[0],loc2[0]]=1
surfactant.iloc[loc2[0],loc1[0]]=1
surfactant=surfactant.set_axis(old_index,axis=0)
surfactant=surfactant.drop(("*"), axis=0)
surfactant=surfactant.drop(("*_"), axis=0)
surfactant=surfactant.drop(("*"), axis=1)
surfactant=surfactant.drop(("*_"), axis=1)
a=list((surfactant.columns))
```

```
        atomic_num_surfactant = []
        atomic_charge=[]
        symbols_surfactant=[]
        atom=[]
        for i in range(len(a)):
            symbols_surfactant.append(Chem.MolFromSmiles("["+ a[i] +"]"))
            for atom in symbols_surfactant[i].GetAtoms():
                atomic_num_surfactant.append(atom.GetAtomicNum())
                atomic_charge.append(atom.GetFormalCharge())
        adjacency_matrix= surfactant.to_numpy()
surfactant_smiles= Chem.MolToSmiles(MolFromGraphs(atomic_num_surfactant, atomic_charge, adjacency_matrix))
surfactant_smiles_mol=Chem.MolFromSmiles(surfactant_smiles)
return adjacency_matrix ,surfactant_smiles, surfactant_smiles_mol
```

"MolFromGraphs" function takes the atomic numbers, charges, and the adjacency matrix as input. The atomic number and formal charge of each atom are computed in the molecule, using the GetAtomicNum() and GetFormalCharge() methods, respectively. Each atom corresponds to the respective row and column labels of the adjacency matrix. These atom-level properties are stored in the atomic_num and atomic_charge lists. It creates an empty editable molecule object (mol) and adds atoms to it based on the number of rows/columns in the adjacency matrix. The function then iterates over the adjacency matrix to add bonds between adjacent atoms in the molecule. Finally, it converts the editable molecule object to a regular molecule object and returns it.

```
def MolFromGraphs(atomic_num, atomic_charge, adjacency_matrix):
    mol = Chem.RWMol()
    node_to_idx = {}
    for i in range(len(atomic_num)):
```

```
        a = Chem.Atom(atomic_num[i])
        a.SetFormalCharge(int(atomic_charge[i]))
        molIdx = mol.AddAtom(a)
        node_to_idx[i] = molIdx
    for ix, row in enumerate(adjacency_matrix):
        for iy, bond in enumerate(row):
            if iy <= ix:
                continue
            if bond == 0:
                continue
            elif bond == 1:
                bond_type = Chem.rdchem.BondType.SINGLE
                mol.AddBond(node_to_idx[ix], node_to_idx[iy], bond_type)
            elif bond == 2:
                bond_type = Chem.rdchem.BondType.DOUBLE
                mol.AddBond(node_to_idx[ix], node_to_idx[iy], bond_type)
            elif bond == 3:
                bond_type = Chem.rdchem.BondType.TRIPLE
                mol.AddBond(node_to_idx[ix], node_to_idx[iy], bond_type)
            elif bond == 1.5:
                bond_type = Chem.rdchem.BondType.AROMATIC
                mol.AddBond(node_to_idx[ix], node_to_idx[iy], bond_type)
    mol = mol.GetMol()
    return mol
```

An example of a tail and head combination, along with the conversion of the surfactant adjacency matrix to SMILE notation by using these functions is provided. Table S1 contains the bond matrix of the tail and Table S2 contains the bond matrix of the head, which serve as inputs to "combination_head_tail" function. Table

S3 presents the bond matrix of the whole surfactant molecule obtained as the first output of that function, and the SMILE notation, which corresponds to the second output is CCCCCCCC=CC(=O)COS(=O)(=O)[O-]. Finally, the surfactant molecule is depicted in Figure S1.

Table S1: Bond matrix of the tail

	*	C	C	C	C	C	C	C	C	C
*	0	0	0	0	0	0	0	1	0	0
C	0	0	1	0	0	0	0	0	1	0
C	0	1	0	1	0	0	0	0	0	0
C	0	0	1	0	1	0	0	0	0	0
C	0	0	0	1	0	1	0	0	0	0
C	0	0	0	0	1	0	1	0	0	0
C	0	0	0	0	0	1	0	0	0	1
C	1	0	0	0	0	0	0	0	2	0
C	0	1	0	0	0	0	0	2	0	0
C	0	0	0	0	0	0	1	0	0	0

Table S2: Bond matrix of the Head

	*	O	S	O	O	O-	C	O	C
*	0	0	0	0	0	0	1	0	0
O	0	0	1	0	0	0	0	0	1
S	0	1	0	2	2	1	0	0	0
O	0	0	2	0	0	0	0	0	0
O	0	0	2	0	0	0	0	0	0
O-	0	0	1	0	0	0	0	0	0
C	1	0	0	0	0	0	0	2	1
O	0	0	0	0	0	0	2	0	0
C	0	1	0	0	0	0	1	0	0

Table S3: Bond matrix of the surfactant molecule

	O	S	O	O	O-	C	O	C	C	C	C	C	C	C	C	C	C
O	0	1	0	0	0	0	0	1	0	0	0	0	0	0	0	0	0

**S**	1	0	2	2	1	0	0	0	0	0	0	0	0	0	0	0	0
**O**	0	2	0	0	0	0	0	0	0	0	0	0	0	0	0	0	0
**O**	0	2	0	0	0	0	0	0	0	0	0	0	0	0	0	0	0
**O-**	0	1	0	0	0	0	0	0	0	0	0	0	0	0	0	0	0
**C**	0	0	0	0	0	0	2	1	0	0	0	0	0	0	1	0	0
**O**	0	0	0	0	0	2	0	0	0	0	0	0	0	0	0	0	0
**C**	1	0	0	0	0	1	0	0	0	0	0	0	0	0	0	0	0
**C**	0	0	0	0	0	0	0	0	0	1	0	0	0	0	0	1	0
**C**	0	0	0	0	0	0	0	0	1	0	1	0	0	0	0	0	0
**C**	0	0	0	0	0	0	0	0	0	1	0	1	0	0	0	0	0
**C**	0	0	0	0	0	0	0	0	0	0	1	0	1	0	0	0	0
**C**	0	0	0	0	0	0	0	0	0	0	0	1	0	1	0	0	0
**C**	0	0	0	0	0	0	0	0	0	0	0	0	1	0	0	0	1
**C**	0	0	0	0	0	1	0	0	0	0	0	0	0	0	0	2	0
**C**	0	0	0	0	0	0	0	0	1	0	0	0	0	0	2	0	0
**C**	0	0	0	0	0	0	0	0	0	0	0	0	0	1	0	0	0

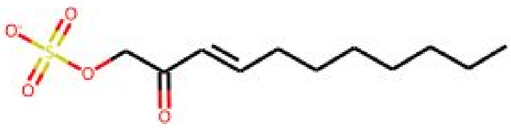

Figure S1: Surfactant molecule structure

GENERATED HEADS

Figures S2 to S103 illustrate the molecular structures of all generated head fragments considered for surfactant design.

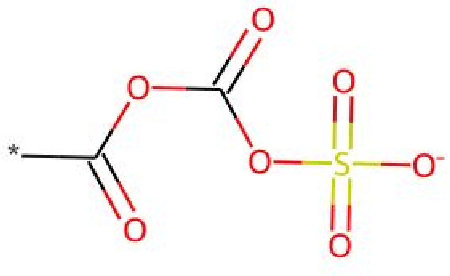

Figure S2: Molecular structure of Head 1: *C(=O)OC(=O)OS(=O)(=O)[O-]

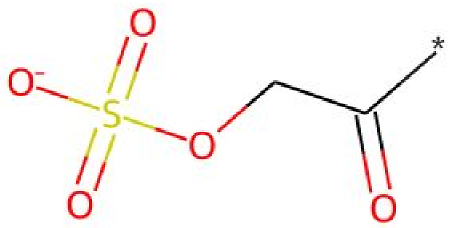

Figure S3: Molecular structure of Head 2: *C(=O)COS(=O)(=O)[O-]

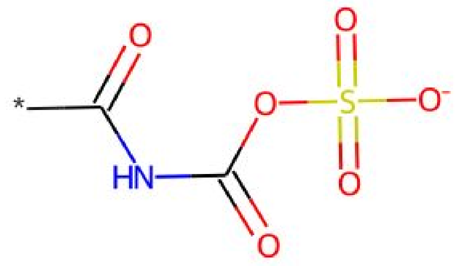

Figure S4: Molecular structure of Head 3: *C(=O)NC(=O)OS(=O)(=O)[O-]

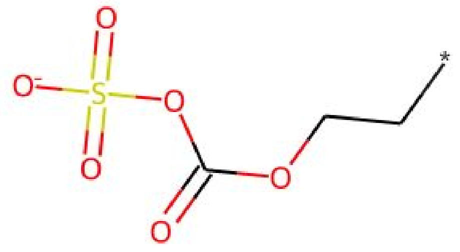

Figure S5: Molecular structure of Head 4: *CCOC(=O)OS(=O)(=O)[O-]

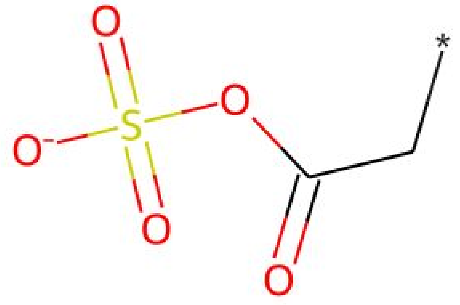

Figure S6: Molecular structure of Head 5: *CC(=O)OS(=O)(=O)[O-]

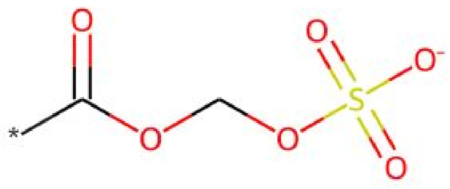

Figure S7: Molecular structure of Head 6: *C(=O)OCOS(=O)(=O)[O-]

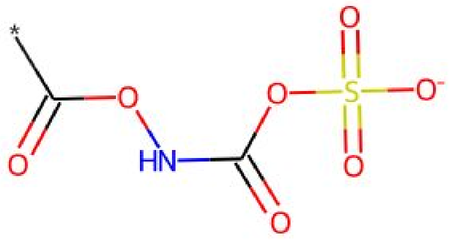

Figure S8: Molecular structure of Head 7: *C(=O)ONC(=O)OS(=O)(=O)[O-]

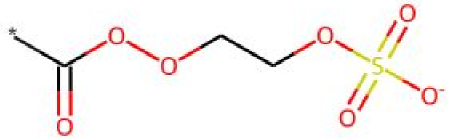

Figure S9: Molecular structure of Head 8: *C(=O)OOCCOS(=O)(=O)[O-]

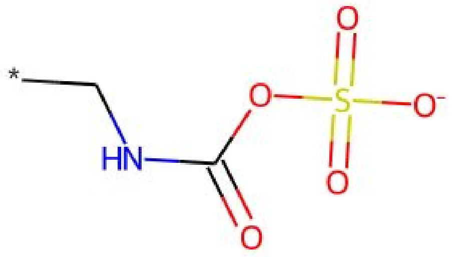

Figure S10: Molecular structure of Head 9: *CNC(=O)OS(=O)(=O)[O-]

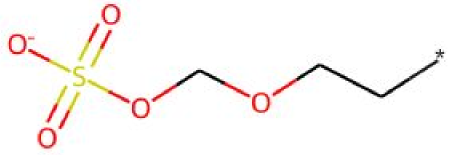

Figure S11: Molecular structure of Head 10: *CCOCOS(=O)(=O)[O-]

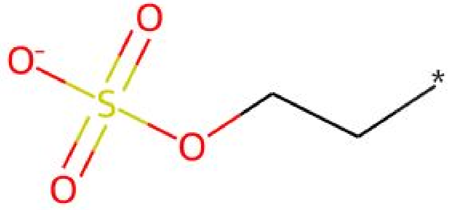

Figure S12: Molecular structure of Head 11: *CCOS(=O)(=O)[O-]

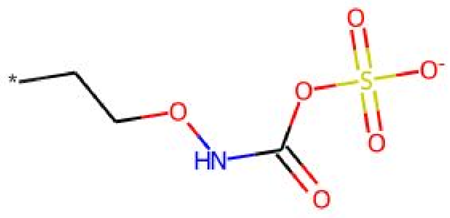

Figure S13: Molecular structure of Head 12: *CCONC(=O)OS(=O)(=O)[O-]

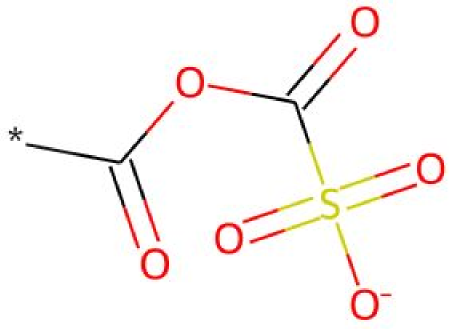

Figure S14: Molecular structure of Head 13: *C(=O)OC(=O)S(=O)(=O)[O-]

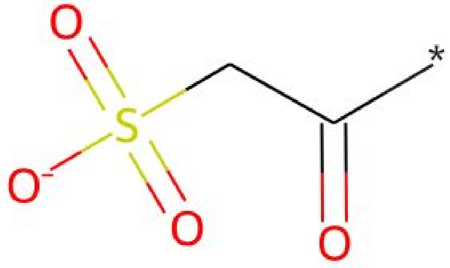

Figure S15: Molecular structure of Head 14: *C(=O)CS(=O)(=O)[O-]

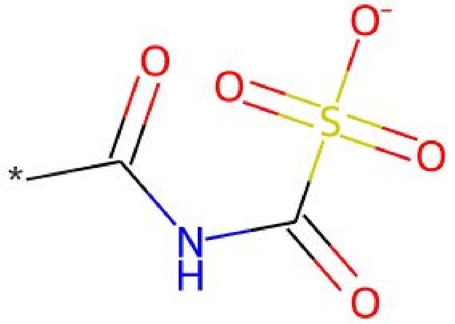

Figure S16: Molecular structure of Head 15: *C(=O)NC(=O)S(=O)(=O)[O-]

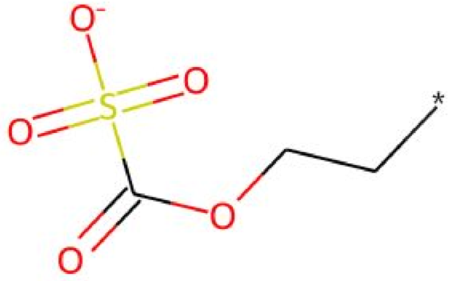

Figure S17: Molecular structure of Head 16: *CCOC(=O)S(=O)(=O)[O-]

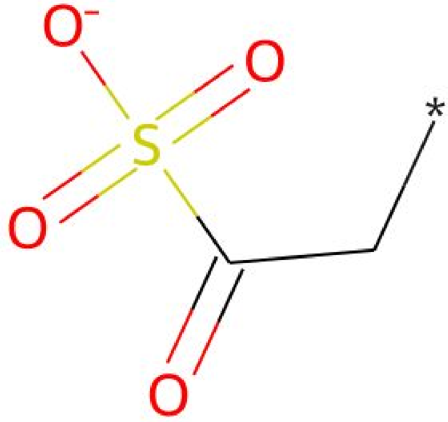

Figure S18: Molecular structure of Head 17: *CC(=O)S(=O)(=O)[O-]

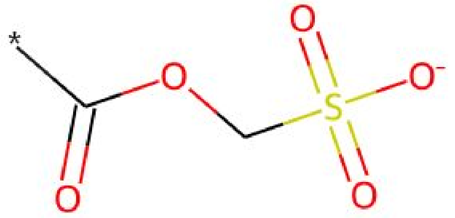

Figure S19: Molecular structure of Head 18: *C(=O)OCS(=O)(=O)[O-]

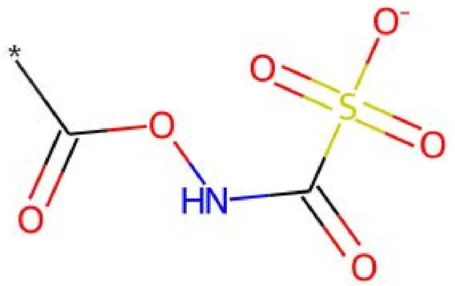

Figure S20: Molecular structure of Head 19: *C(=O)ONC(=O)S(=O)(=O)[O-]

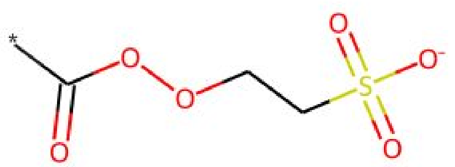

Figure S21: Molecular structure of Head 20: *C(=O)OOCCS(=O)(=O)[O-]

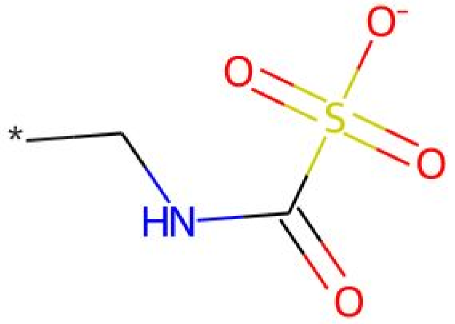

Figure S22: Molecular structure of Head 21: *CNC(=O)S(=O)(=O)[O-]

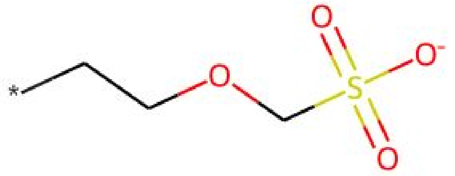

Figure S23: Molecular structure of Head 22: *CCOCS(=O)(=O)[O-]

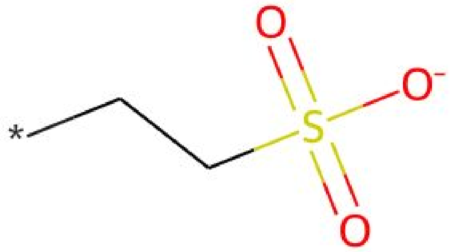

Figure S24: Molecular structure of Head 23: *CCS(=O)(=O)[O-]

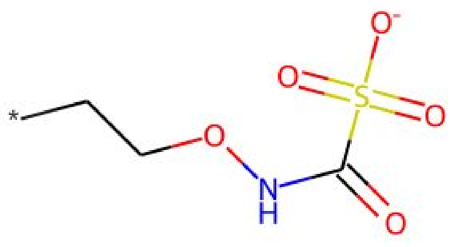

Figure S25: Molecular structure of Head 24: *CCONC(=O)S(=O)(=O)[O-]

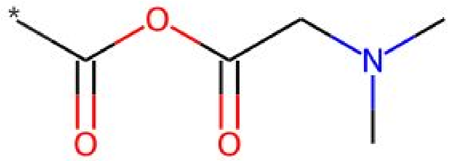

Figure S26: Molecular structure of Head 25: *C(=O)OC(=O)CN(C)C

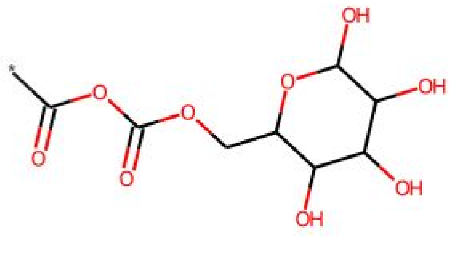

Figure S27: Molecular structure of Head 26: *C(=O)OC(=O)OCC1OC(O)C(O)C(O)C1O

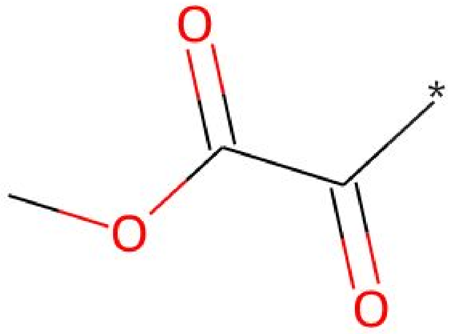

Figure S28: Molecular structure of Head 27: *C(=O)C(=O)OC

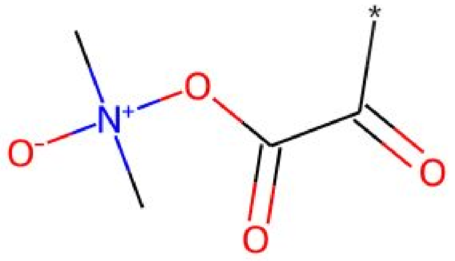

Figure S29: Molecular structure of Head 28: *C(=O)C(=O)O[N+](C)(C)[O-]

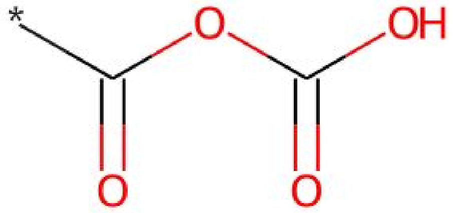

Figure S30: Molecular structure of Head 29: *C(=O)OC(=O)O

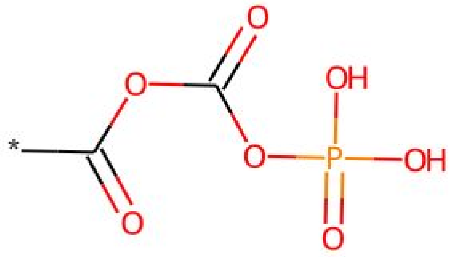

Figure S31: Molecular structure of Head 30: *C(=O)OC(=O)OP(=O)(O)O

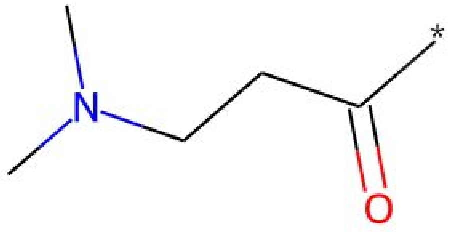

Figure S32: Molecular structure of Head 31: *C(=O)CCN(C)C

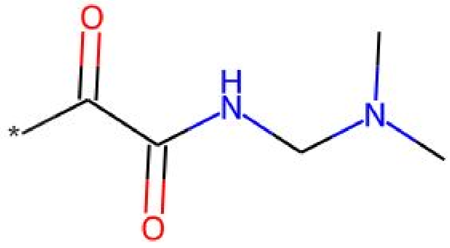

Figure S33: Molecular structure of Head 32: *C(=O)C(=O)NCN(C)C

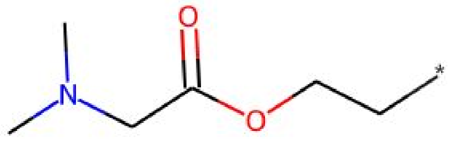

Figure S34: Molecular structure of Head 33: *CCOC(=O)CN(C)C

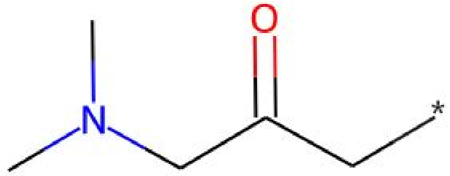

Figure S35: Molecular structure of Head 34: *CC(=O)CN(C)C

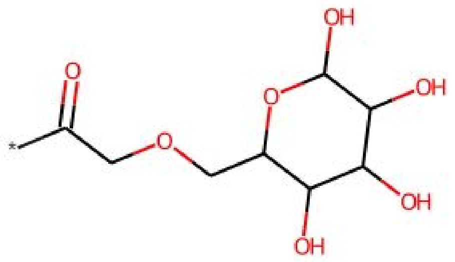

Figure S36: Molecular structure of Head 35: *C(=O)COCC1OC(O)C(O)C(O)C1O

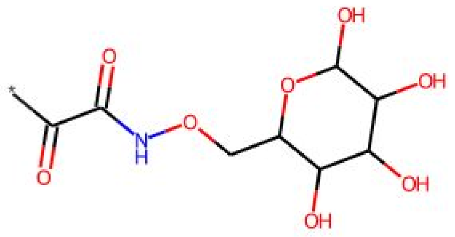

Figure S37: Molecular structure of Head 36: *C(=O)C(=O)NOCC1OC(O)C(O)C(O)C1O

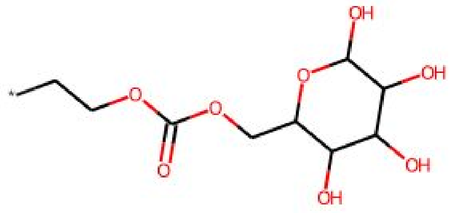

Figure S38: Molecular structure of Head 37: *CCOC(=O)OCC1OC(O)C(O)C(O)C1O

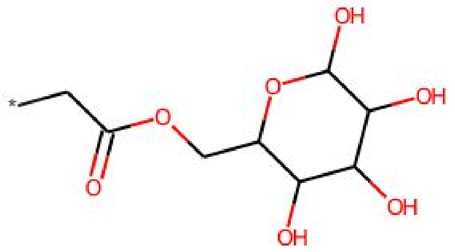

Figure S39: Molecular structure of Head 38: *CC(=O)OCC1OC(O)C(O)C(O)C1O

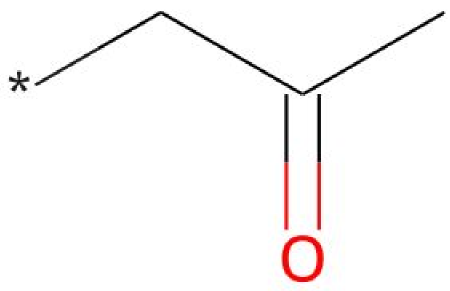

Figure S40: Molecular structure of Head 39: *CC(C)=O

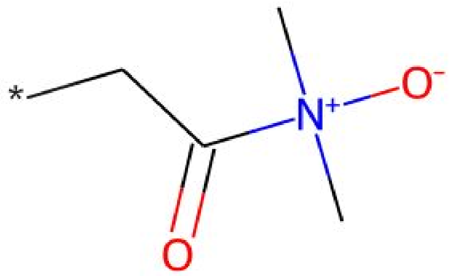

Figure S41: Molecular structure of Head 40: *CC(=O)[N+](C)(C)[O-]

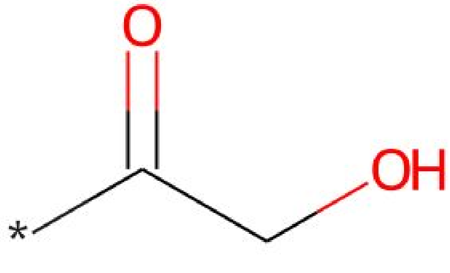

Figure S42: Molecular structure of Head 41: *C(=O)CO

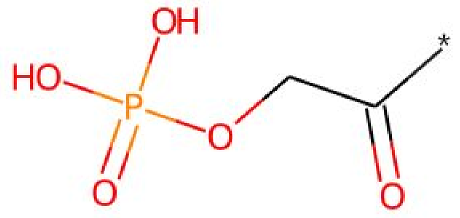

Figure S43: Molecular structure of Head 42: *C(=O)COP(=O)(O)O

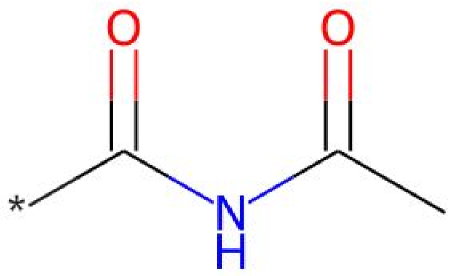

Figure S44: Molecular structure of Head 43: *C(=O)NC(C)=O

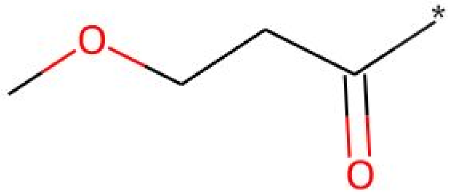

Figure S45: Molecular structure of Head 44: *C(=O)CCOC

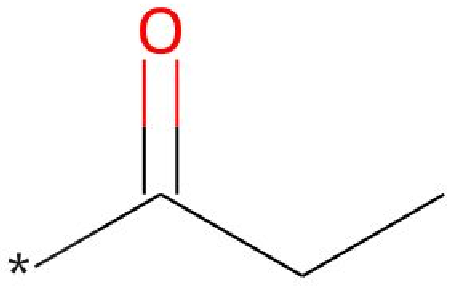

Figure S46: Molecular structure of Head 45: *C(=O)CC

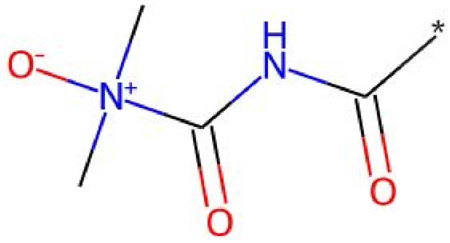

Figure S47: Molecular structure of Head 46: *C(=O)NC(=O)[N+](C)(C)[O-]

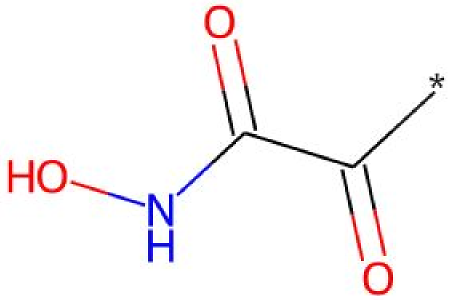

Figure S48: Molecular structure of Head 47: *C(=O)C(=O)NO

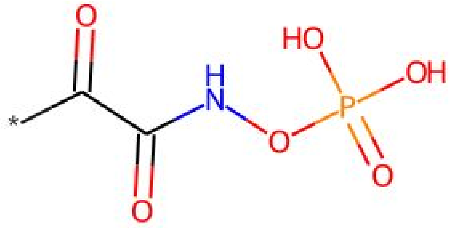

Figure S49: Molecular structure of Head 48: *C(=O)C(=O)NOP(=O)(O)O

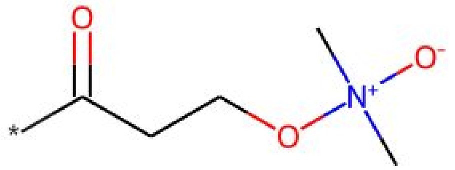

Figure S50: Molecular structure of Head 49: *C(=O)CCO[N+](C)(C)[O-]

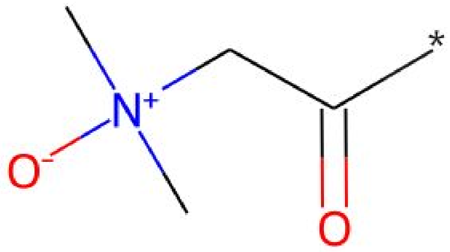

Figure S51: Molecular structure of Head 50: *C(=O)C[N+](C)(C)[O-]

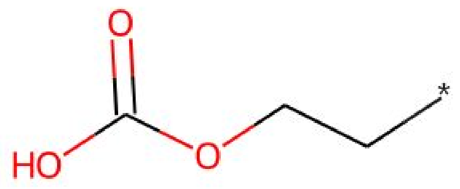

Figure S52: Molecular structure of Head 51: *CCOC(=O)O

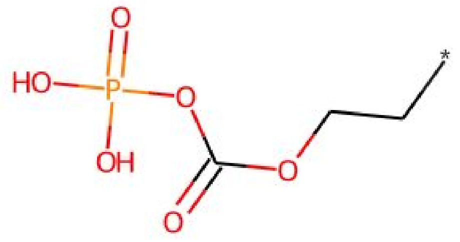

Figure S53: Molecular structure of Head 52: *CCOC(=O)OP(=O)(O)O

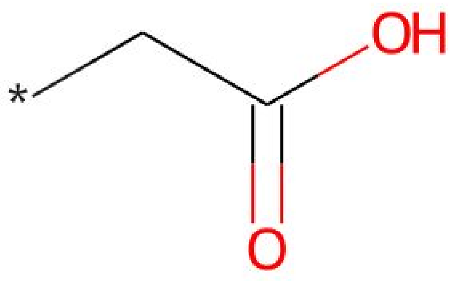

Figure S54: Molecular structure of Head 53: *CC(=O)O

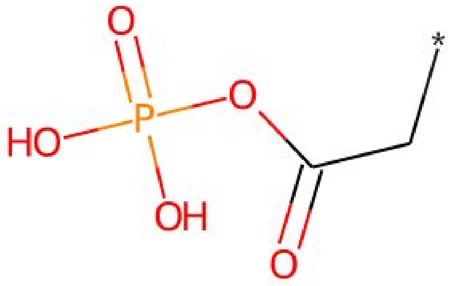

Figure S55: Molecular structure of Head 54: *CC(=O)OP(=O)(O)O

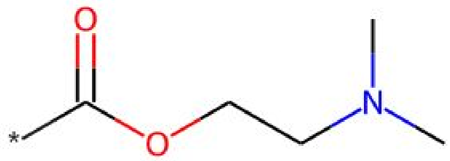

Figure S56: Molecular structure of Head 55: *C(=O)OCCN(C)C

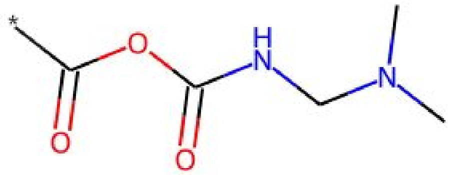

Figure S57: Molecular structure of Head 56: *C(=O)OC(=O)NCN(C)C

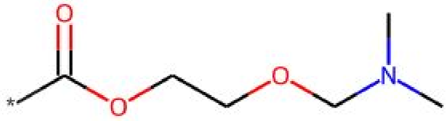

Figure S58: Molecular structure of Head 57: *C(=O)OCCOCN(C)C

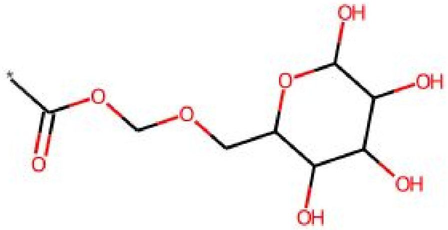

Figure S59: Molecular structure of Head 58: *C(=O)OCOCC1OC(O)C(O)C(O)C1O

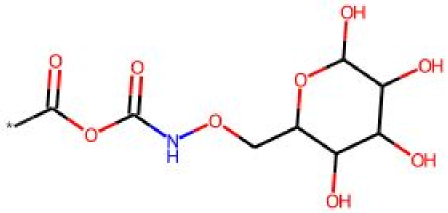

Figure S60: Molecular structure of Head 59: *C(=O)OC(=O)NOCC1OC(O)C(O)C(O)C1O

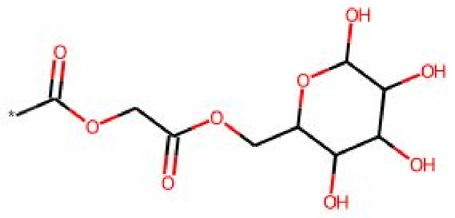

Figure S61: Molecular structure of Head 60: *C(=O)OCC(=O)OCC1OC(O)C(O)C(O)C1O

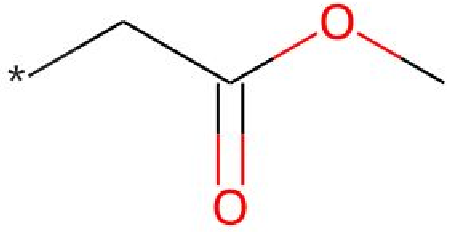

Figure S62: Molecular structure of Head 61: *CC(=O)OC

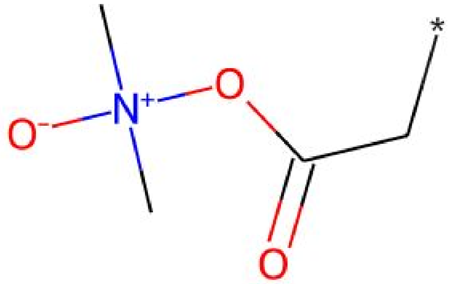

Figure S63: Molecular structure of Head 62: *CC(=O)O[N+](C)(C)[O-]

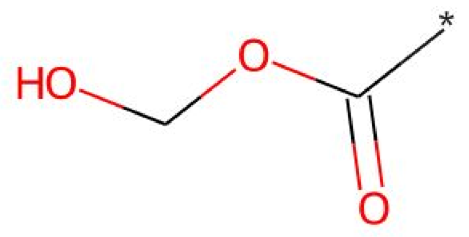

Figure S64: Molecular structure of Head 63: *C(=O)OCO

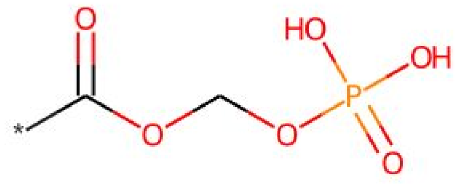

Figure S65: Molecular structure of Head 64: *C(=O)OCOP(=O)(O)O

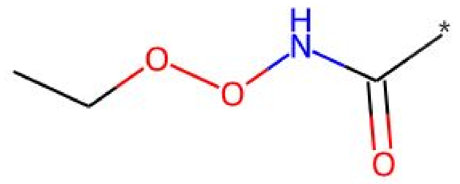

Figure S66: Molecular structure of Head 65: *C(=O)NOOCC

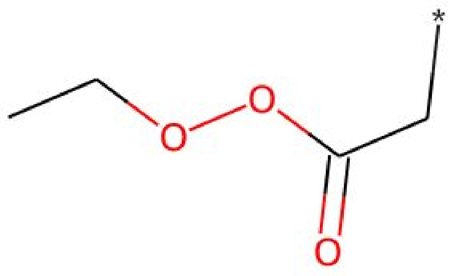

Figure S67: Molecular structure of Head 66: *CC(=O)OOCC

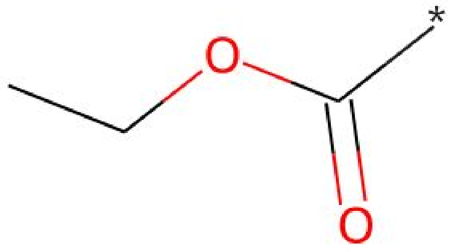

Figure S68: Molecular structure of Head 67: *C(=O)OCC

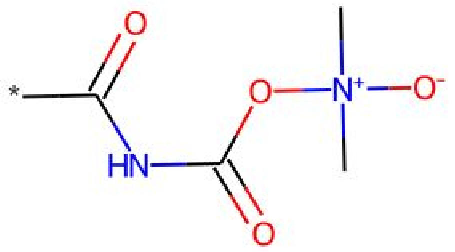

Figure S69: Molecular structure of Head 68: *C(=O)NC(=O)O[N+](C)(C)[O-]

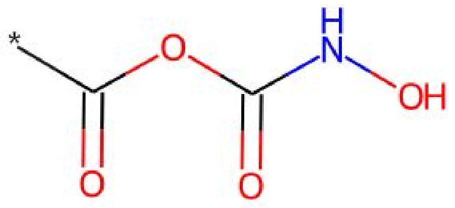

Figure S70: Molecular structure of Head 69: *C(=O)OC(=O)NO

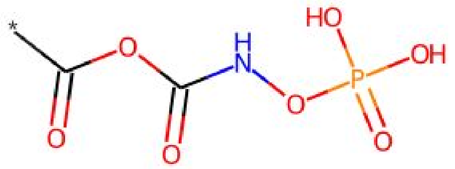

Figure S71: Molecular structure of Head 70: *C(=O)OC(=O)NOP(=O)(O)O

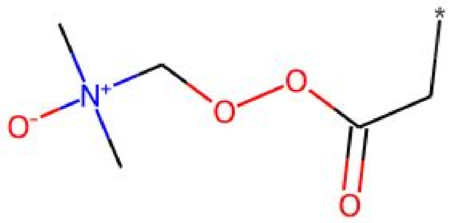

Figure S72: Molecular structure of Head 71: *CC(=O)OOC[N+](C)(C)[O-]

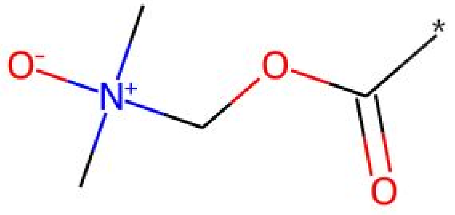

Figure S73: Molecular structure of Head 72: *C(=O)OC[N+](C)(C)[O-]

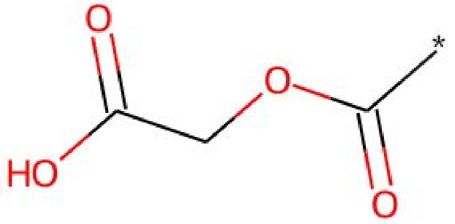

Figure S74: Molecular structure of Head 73: *C(=O)OCC(=O)O

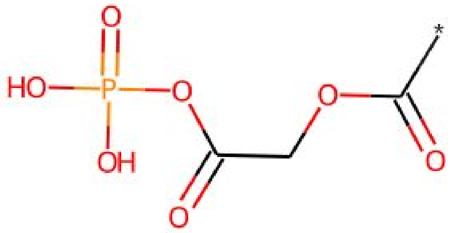

Figure S75: Molecular structure of Head 74: *C(=O)OCC(=O)OP(=O)(O)O

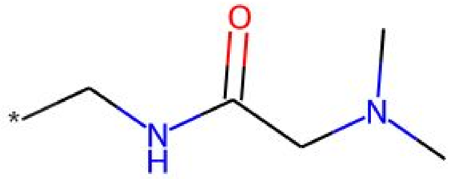

Figure S76: Molecular structure of Head 75: *CNC(=O)CN(C)C

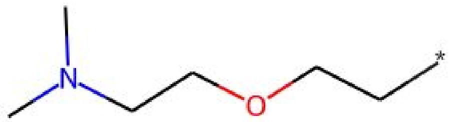

Figure S77: Molecular structure of Head 76: *CCOCCN(C)C

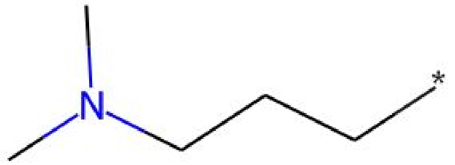

Figure S78: Molecular structure of Head 77: *CCCN(C)C

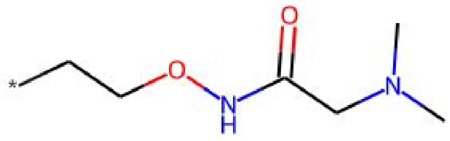

Figure S79: Molecular structure of Head 78: *CCONC(=O)CN(C)C

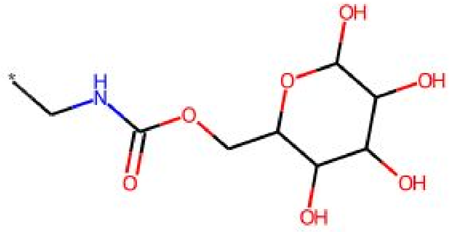

Figure S80: Molecular structure of Head 79: *CNC(=O)OCC1OC(O)C(O)C(O)C1O

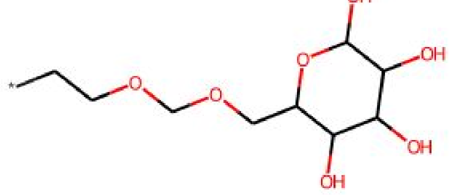

Figure S81: Molecular structure of Head 80: *CCOCOCC1OC(O)C(O)C(O)C1O

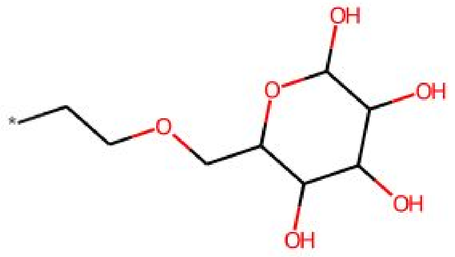

Figure S82: Molecular structure of Head 81: *CCOCC1OC(O)C(O)C(O)C1O

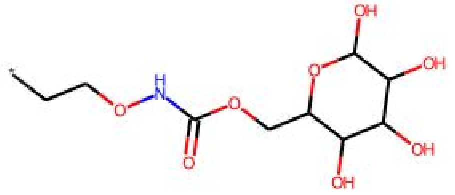

Figure S83: Molecular structure of Head 82: *CCONC(=O)OCC1OC(O)C(O)C(O)C1O

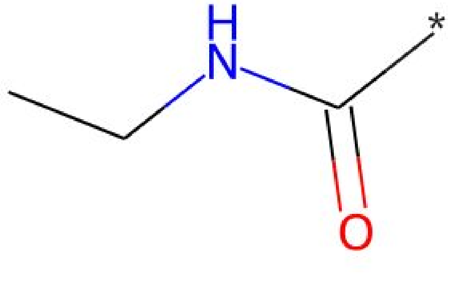

Figure S84: Molecular structure of Head 83: *C(=O)NCC

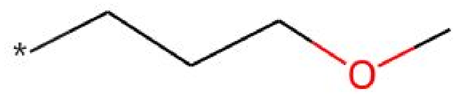

Figure S85: Molecular structure of Head 84: *CCCOC

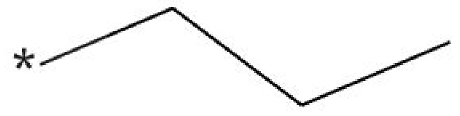

Figure S86: Molecular structure of Head 85: *CCC

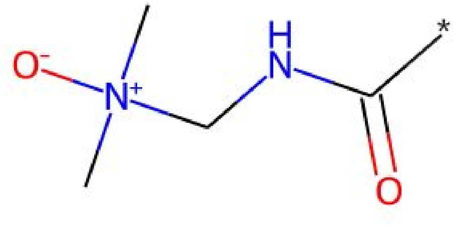

Figure S87: Molecular structure of Head 86: *C(=O)NC[N+](C)(C)[O-]

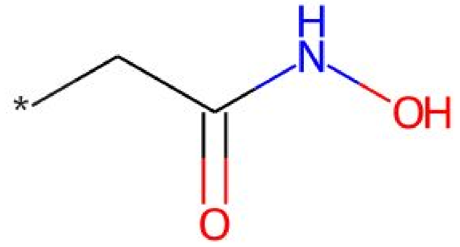

Figure S88: Molecular structure of Head 87: *CC(=O)NO

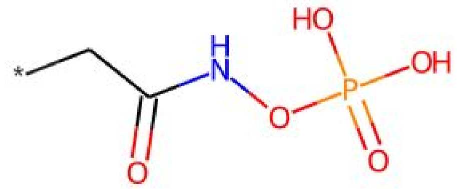

Figure S89: Molecular structure of Head 88: *CC(=O)NOP(=O)(O)O

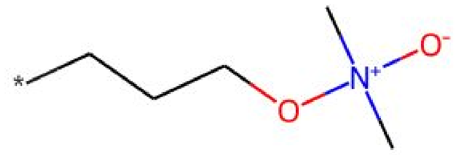

Figure S90: Molecular structure of Head 89: *CCCO[N+](C)(C)[O-]

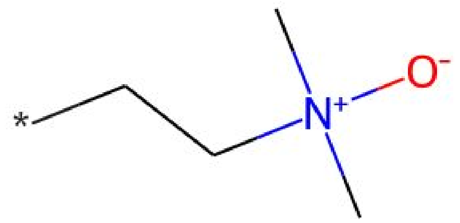

Figure S91: Molecular structure of Head 90: *CC[N+](C)(C)[O-]

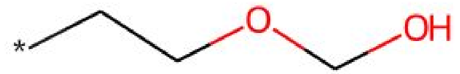

Figure S92: Molecular structure of Head 91: *CCOCO

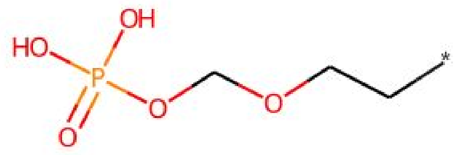

Figure S93: Molecular structure of Head 92: *CCOCOP(=O)(O)O

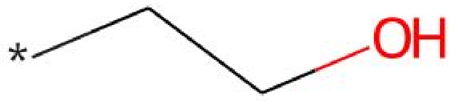

Figure S94: Molecular structure of Head 93: *CCO

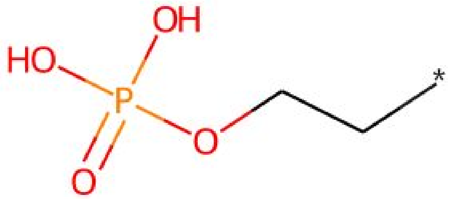

Figure S95: Molecular structure of Head 94: *CCOP(=O)(O)O

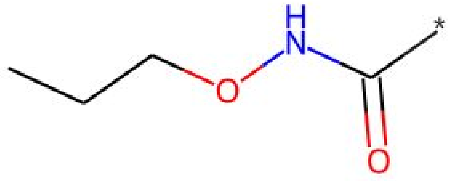

Figure S96: Molecular structure of Head 95: *C(=O)NOCCC

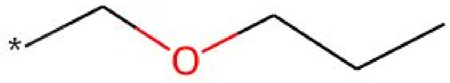

Figure S97: Molecular structure of Head 96: *COCCC

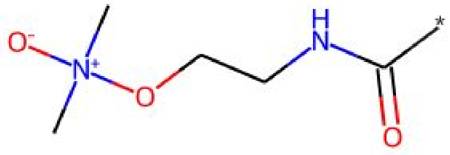

Figure S98: Molecular structure of Head 97: *C(=O)NCCO[N+](C)(C)[O-]

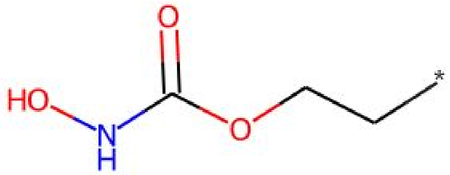

Figure S99: Molecular structure of Head 98: *CCOC(=O)NO

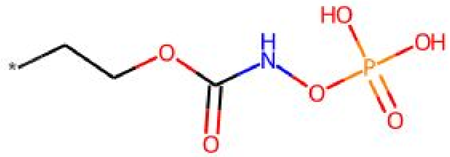

Figure S100: Molecular structure of Head 99: *CCOC(=O)NOP(=O)(O)O

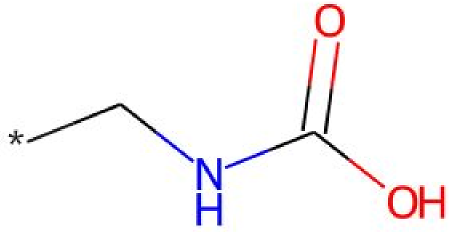

Figure S101: Molecular structure of Head 100: *CNC(=O)O

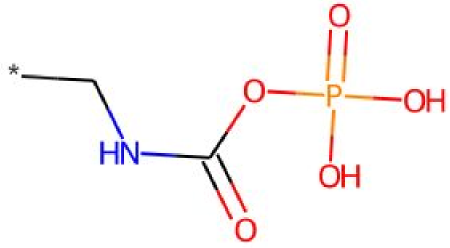

Figure S102: Molecular structure of Head 101: *CNC(=O)OP(=O)(O)O

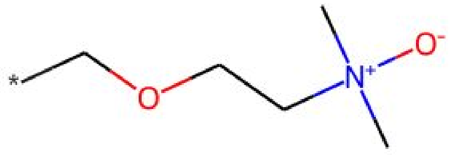

Figure S103: Molecular structure of Head 102: *COCC[N+](C)(C)[O-]

GENERATED TAILS

Figures S104 to S153 illustrate the molecular structures of all generated tail fragments considered for surfactant design.

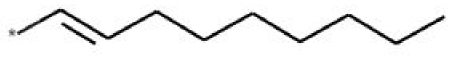

Figure S104: Molecular structure of Tail 1: *C=CCCCCCCC

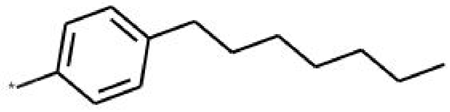

Figure S105: Molecular structure of Tail 2: *c1ccc(CCCCCCC)cc1

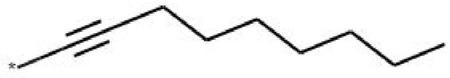

Figure S106: Molecular structure of Tail 3: *C#CCCCCCC

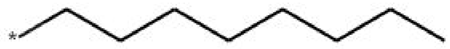

Figure S107: Molecular structure of Tail 4: *CCCCCCCC

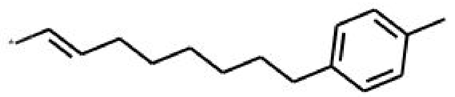

Figure S108: Molecular structure of Tail 5: *C=CCCCCCCc1ccc(C)cc1

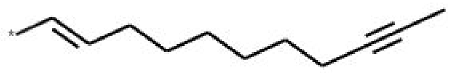

Figure S109: Molecular structure of Tail 6: *C=CCCCCCCC#CC

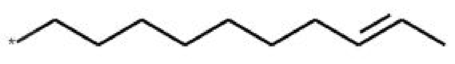

Figure S110: Molecular structure of Tail 7: *CCCCCCCC=CC

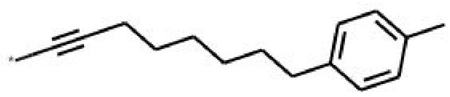

Figure S111: Molecular structure of Tail 8: *C#CCCCCCCc1ccc(C)cc1

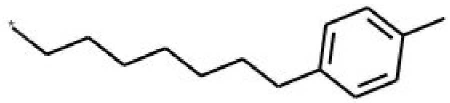

Figure S112: Molecular structure of Tail 9: *CCCCCCCc1ccc(C)cc1

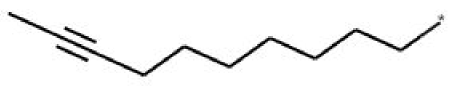

Figure S113: Molecular structure of Tail 10: *CCCCCCCC#CC

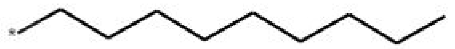

Figure S114: Molecular structure of Tail 11: *CCCCCCCCC

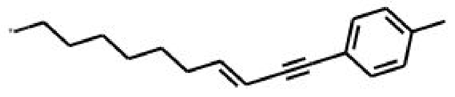

Figure S115: Molecular structure of Tail 12: *CCCCCCC=CC#Cc1ccc(C)cc1

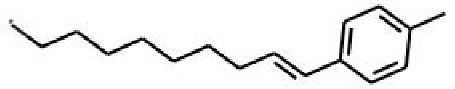

Figure S116: Molecular structure of Tail 13: *CCCCCCCC=Cc1ccc(C)cc1

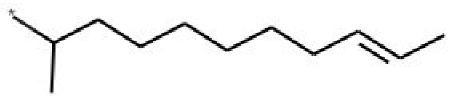

Figure S117: Molecular structure of Tail 14: *C(C)CCCCCCC=CC

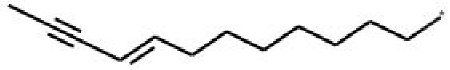

Figure S118: Molecular structure of Tail 15: *CCCCCCCC=CC#CC

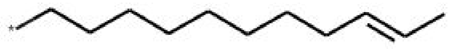

Figure S119: Molecular structure of Tail 16: *CCCCCCCCC=CC

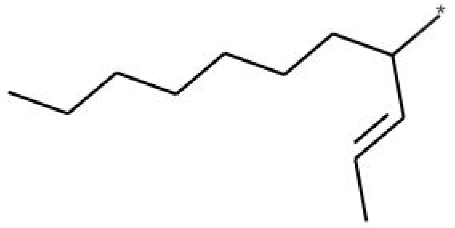

Figure S120: Molecular structure of Tail 17: *C(C=CC)CCCCCCC

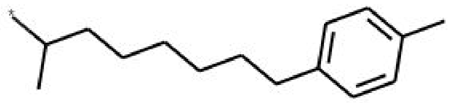

Figure S121: Molecular structure of Tail 18: *C(C)CCCCCCc1ccc(C)cc1

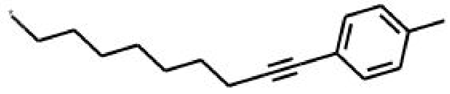

Figure S122: Molecular structure of Tail 19: *CCCCCCCC#Cc1ccc(C)cc1

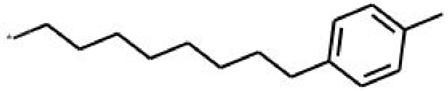

Figure S123: Molecular structure of Tail 20: *CCCCCCCCc1ccc(C)cc1

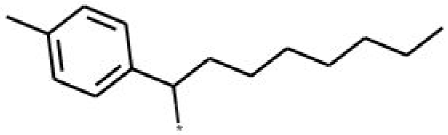

Figure S124: Molecular structure of Tail 21: *C(CCCCCCC)c1ccc(C)cc1

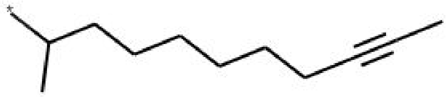

Figure S125: Molecular structure of Tail 22: *C(C)CCCCCCC#CC

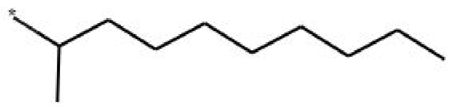

Figure S126: Molecular structure of Tail 23: *C(C)CCCCCCCC

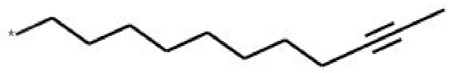

Figure S127: Molecular structure of Tail 24: *CCCCCCCCC#CC

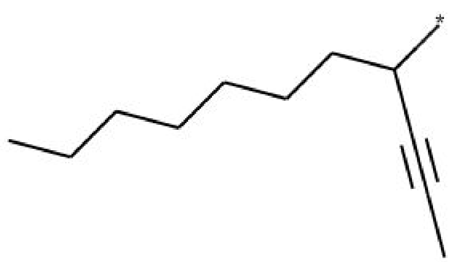

Figure S128: Molecular structure of Tail 25: *C(C#CC)CCCCCCC

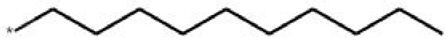

Figure S129: Molecular structure of Tail 26: *CCCCCCCCCC

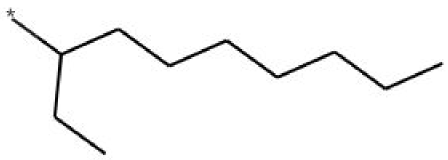

Figure S130: Molecular structure of Tail 27: *C(CC)CCCCCCC

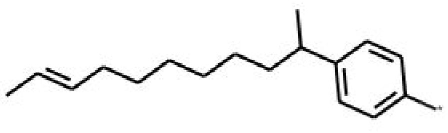

Figure S131: Molecular structure of Tail 28: *c1ccc(C(C)CCCCCCC=CC)cc1

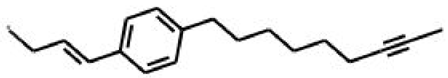

Figure S132: Molecular structure of Tail 29: *CC=Cc1ccc(CCCCCCC#CC)cc1

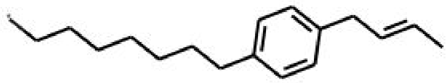

Figure S133: Molecular structure of Tail 30: *CCCCCCCc1ccc(CC=CC)cc1

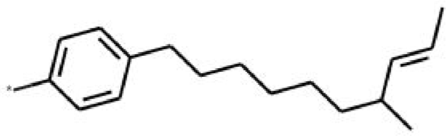

Figure S134: Molecular structure of Tail 31: *c1ccc(CCCCCCC(C)C=CC)cc1

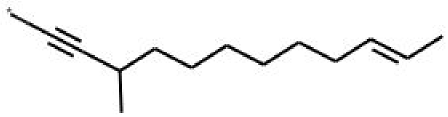

Figure S135: Molecular structure of Tail 32: *C#CC(C)CCCCCCC=CC

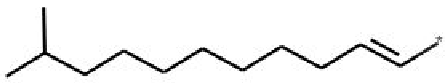

Figure S136: Molecular structure of Tail 33: *C=CCCCCCCCC(C)C

Figure S137: Molecular structure of Tail 34: *CCCCCCCC#CCC=CC

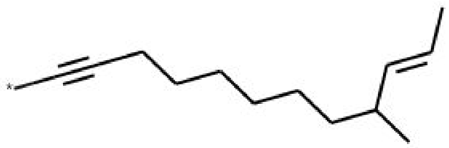

Figure S138: Molecular structure of Tail 35: *C#CCCCCCCC(C)C=CC

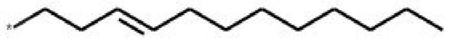

Figure S139: Molecular structure of Tail 36: *CCC=CCCCCCCCC

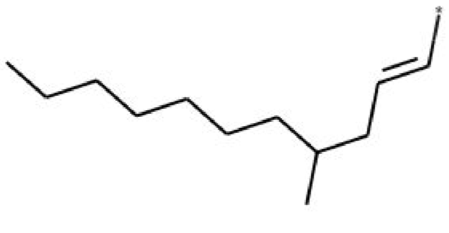

Figure S140: Molecular structure of Tail 37: *C=CCC(C)CCCCCCC

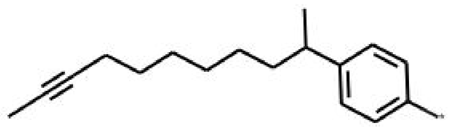

Figure S141: Molecular structure of Tail 38: *c1ccc(C(C)CCCCCCC#CC)cc1

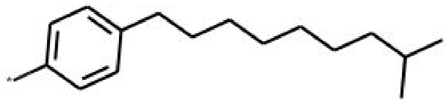

Figure S142: Molecular structure of Tail 39: *c1ccc(CCCCCCCC(C)C)cc1

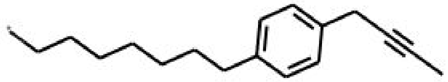

Figure S143: Molecular structure of Tail 40: *CCCCCCCc1ccc(CC#CC)cc1

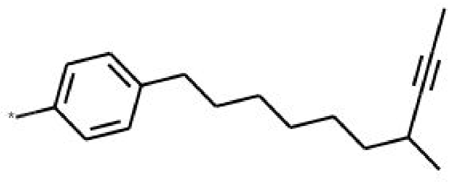

Figure S144: Molecular structure of Tail 41: *c1ccc(CCCCCCC(C)C#CC)cc1

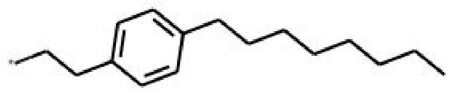

Figure S145: Molecular structure of Tail 42: *CCc1ccc(CCCCCCCC)cc1

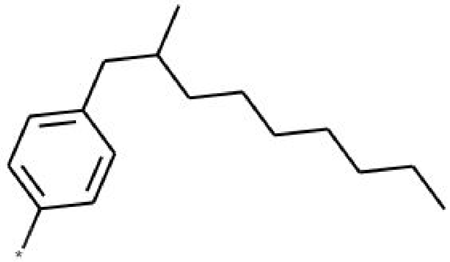

Figure S146: Molecular structure of Tail 43: *c1ccc(CC(C)CCCCCCC)cc1

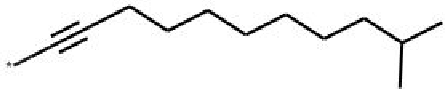

Figure S147: Molecular structure of Tail 44: *C#CCCCCCCCC(C)C

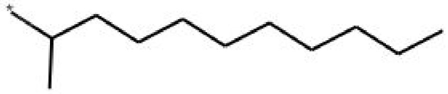

Figure S148: Molecular structure of Tail 45: *C(C)CCCCCCCCC

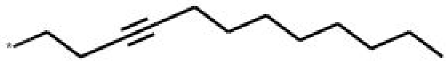

Figure S150: Molecular structure of Tail 47: *CCC#CCCCCCCCC

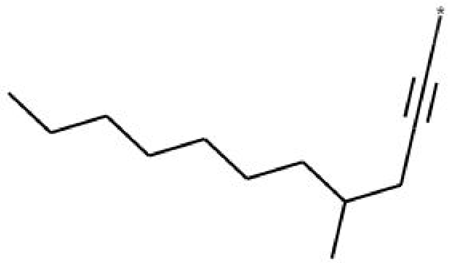

Figure S151: Molecular structure of Tail 48: *C#CCC(C)CCCCCCC

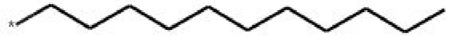

Figure S152: Molecular structure of Tail 49: *CCCCCCCCCCC

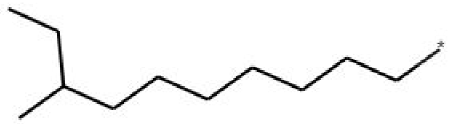

Figure S153: Molecular structure of Tail 50: *CCCCCCCC(C)CC
